# E3 ubiquitin ligase rififylin has yin and yang effects on rabbit cardiac transient outward potassium currents (*I*_to_) and corresponding channel proteins

**DOI:** 10.1016/j.jbc.2024.105759

**Published:** 2024-02-15

**Authors:** Anatoli Y. Kabakov, Karim Roder, Peter Bronk, Nilüfer N. Turan, Saroj Dhakal, Mingwang Zhong, Yichun Lu, Zachary A. Zeltzer, Yonatan B. Najman-Licht, Alain Karma, Gideon Koren

**Affiliations:** 1 Division of Cardiology, Department of Medicine, Cardiovascular Research Center, Rhode Island Hospital, The Warren Alpert Medical School, Brown University, Providence, Rhode Island, USA; 2 Physics Department and Center for Interdisciplinary Research on Complex Systems, Northeastern University, Boston, Massachusetts, USA

**Keywords:** cardiomyocyte, ubiquitin ligase, action potential duration, RFFL, Kv1.4, Kv4.3, KChIP2, potassium channel

## Abstract

Genome-wide association studies have reported a correlation between a SNP of the RING finger E3 ubiquitin protein ligase rififylin (*RFFL*) and QT interval variability in humans (Newton-Cheh *et al*., 2009). Previously, we have shown that RFFL downregulates expression and function of the human-like ether-a-go-go-related gene potassium channel and corresponding rapidly activating delayed rectifier potassium current (*I*_Kr_) in adult rabbit ventricular cardiomyocytes. Here, we report that RFFL also affects the transient outward current (*I*_to_), but in a peculiar way. RFFL overexpression in adult rabbit ventricular cardiomyocytes significantly decreases the contribution of its fast component (*I*_to,f_) from 35% to 21% and increases the contribution of its slow component (*I*_to,s_) from 65% to 79%. Since *I*_to,f_ in rabbits is mainly conducted by Kv4.3, we investigated the effect of RFFL on Kv4.3 expressed in HEK293A cells. We found that RFFL overexpression reduced Kv4.3 expression and corresponding *I*_to,f_ in a RING domain–dependent manner in the presence or absence of its accessory subunit Kv channel-interacting protein 2. On the other hand, RFFL overexpression in Kv1.4-expressing HEK cells leads to an increase in both Kv1.4 expression level and *I*_to,s_, similarly in a RING domain–dependent manner. Our physiologically detailed rabbit ventricular myocyte computational model shows that these yin and yang effects of RFFL overexpression on *I*_to,f,_ and *I*_to,s_ affect phase 1 of the action potential waveform and slightly decrease its duration in addition to suppressing *I*_Kr_. Thus, RFFL modifies cardiac repolarization reserve *via* ubiquitination of multiple proteins that differently affect various potassium channels and cardiac action potential duration.

The QT interval is an important diagnostic feature on surface electrocardiograms because it reflects the duration of the ventricular action potential. Genome-wide association studies have provided evidence for the association of the human cardiac QT interval with a genetic variant near the gene encoding the E3 ubiquitin (Ub) ligase RING finger and FYVE-like domain–containing protein (*RFFL*) on chromosome 17q12 (1, 2, 3). Ubiquitylation (also known as ubiquitination) targets most proteins for degradation by the 26S proteasome. Other functions of ubiquitination include internalization and lysosomal targeting, modulation of protein interactions, alteration of subcellular distribution, regulation of transcription, DNA repair, propagation of transmembrane signaling, and antiviral immune response (4, 5). Ubiquitination occurs through the sequential action of three classes of protein: Ub-activating enzymes (E1s), Ub-conjugating enzymes (E2s), and Ub-protein ligases (E3s).

There are multiple targets of the RFFL E3 Ub ligase. RFFL is involved in endocytic trafficking, which is regulated by ubiquitination of cargoes and endocytic machineries. An RFFL dominant–negative mutant induced clustering of endocytic recycling compartments and delayed endocytic cargo recycling (6). RFFL also contributes to tumorigenesis by repressing caspases and tumor suppressor genes (7). Of note, upregulation of RFFL leads to ubiquitination and degradation of the PRR5L subunit of mammalian target of rapamycin complex 2 (8). Importantly, RFFL is responsible for chaperone-independent ubiquitination of misfolded cystic fibrosis transmembrane conductance regulator (CFTR) (9), since the most common CFTR mutant, ΔF508-CFTR, is removed from the plasma membrane for lysosomal degradation by ubiquitination. Excess of RFFL also inhibits recycling from the endocytic recycling compartment (10), indirectly affects endocytosis, and enhances intracellular protein polyubiquitination (11).

To clarify the effects of RFFL on the QT interval and specifically on the repolarization reserve in larger animals, we initially studied how RFFL affects the major repolarizing current in large mammals, specifically the rapidly activating delayed rectifier potassium current (*I*_Kr_) in adult rabbit ventricular cardiomyocytes (ARbCMs). We have found that RFFL polyubiquitinates the corresponding human-like ether-a-go-go-related gene (hERG) potassium channel in the endoplasmic reticulum (ER), leading to proteasomal degradation of hERG and to an almost complete disappearance of *I*_Kr._ This activity of RFFL depended on an intact RING domain, which is necessary for Ub ligase activity. Loss of hERG reduces repolarization reserve and, based on computational studies, increases the QT interval (12). Surprisingly, RFFL has a negative correlation coefficient with mouse cardiac hypertrophy caused by a 3-week isoproterenol treatment (13). Of note, it has been found that a congenic strain of Dahl salt-sensitive rat with a genomic segment encoding RFFL from the normotensive Lewis rat had significantly higher expression levels of both *RFFL* mRNA and protein, correlating with a significantly shorter QT interval than the original Dahl salt-sensitive rats (14). This indicates that the increase in RFFL likely leads to a shortening of the action potential duration (APD) in rat ventricular myocytes, where the major repolarizing current is not *I*_Kr_, but the transient outward potassium current (*I*_to_). Therefore, to further clarify the effects of RFFL on the QT interval in the rat and larger animals, we studied RFFL effects on *I*_to_ in rabbit cardiomyocytes and on corresponding channel proteins expressed in human embryonic kidney (HEK) cells. We have found that RFFL overexpression decreases the fast component (*I*_to,f_) and increases the slow component (*I*_to,s_) of *I*_to_ in rabbit myocytes. This result correlates with RFFL-induced changes in HEK cells expressing Kv4.3 and Kv1.4 alpha subunits of the corresponding channel proteins. In combination with our previously published findings (12), this article explains the opposite effects of RFFL on QT interval in rodents and larger mammals.

## Results

### RFFL overexpression reduces *I*_to,f_ and increases *I*_to,s_ in ARbCMs

**Figure 1:**
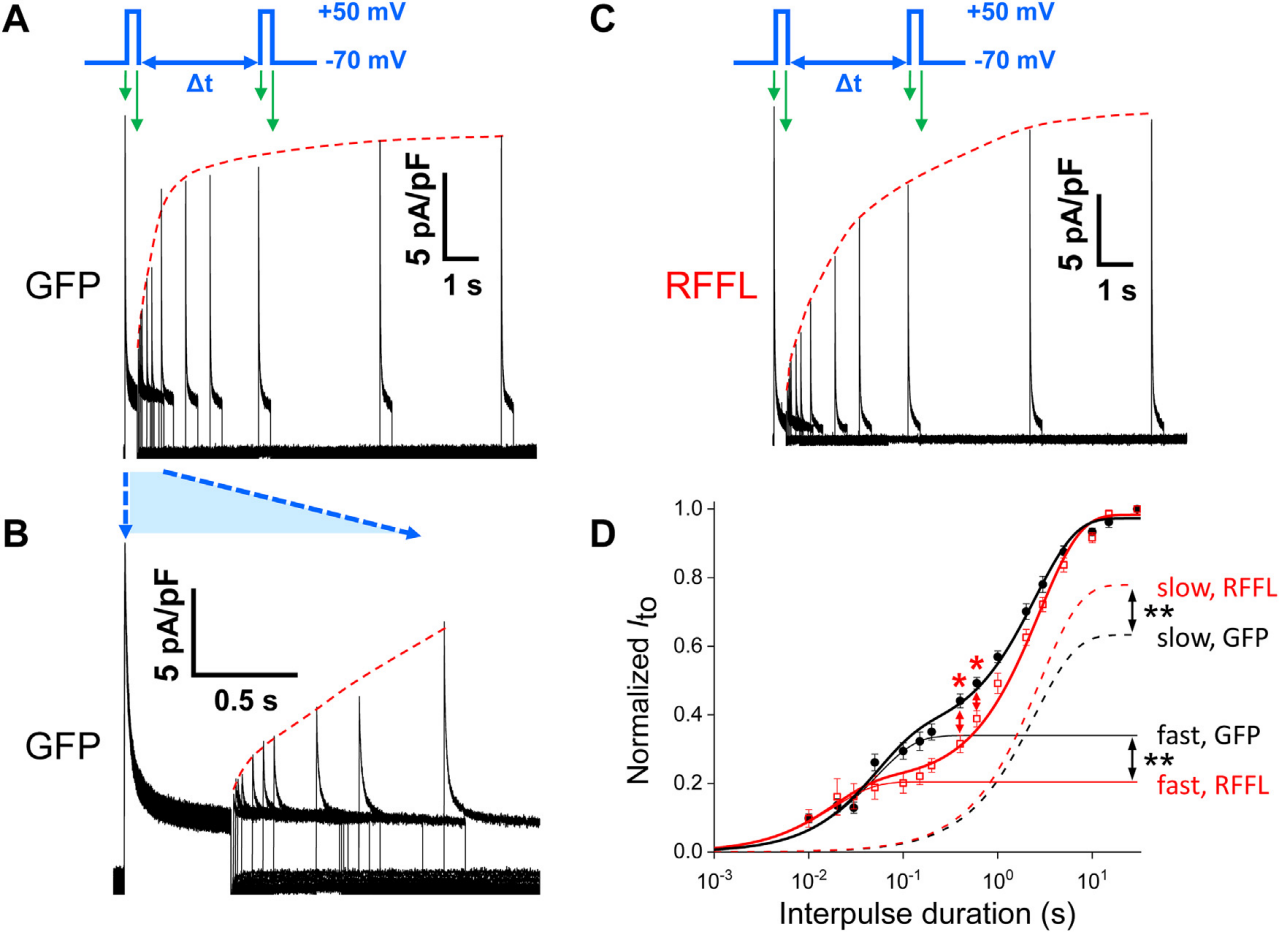
**RFFL overexpression reduces *I***_**to,f**_**but increases *I***_**to,s**_**in ARbCMs.***A*, overlapping representative traces of *I*_to_ in ARbCMs adenovirally expressing GFP obtained with double-pulse protocol from a holding potential of −70 mV to 0.5 s test pulses of +50 mV. The second pulse was applied after a varied recovery interval (Δt) from 10 ms to 15 s. The voltage protocol in the insert is an example of the interpulse time of 5 s with *short green arrows* corresponding to the beginning of the first and second *I*_to_ stimulations and the *long green arrows* corresponding to the ends of the stimulating pulses. The *red dashed curve* reveals a trend of *I*_to_ recovery for this experiment. *B*, *I*_to_ traces shown in (*A*) are zoomed in to show *I*_to_ peaks at shorter recovery intervals. *C*, overlapping representative traces of *I*_to_ in ARbCMs adenovirally expressing RFFL obtained with the same protocol as in (*A*). *D*, the normalized *I*_to_ recovery data (mean ± SEM) of GFP-expressing (N = 5, n = 12) and RFFL-expressing (N = 5, n = 10) ARbCMs are significantly different when compared by a mixed effects model for repeated measures data test (*p* < 0.05). The differences between GFP and RFFL curves are also significant at recovery time points of 400 and 600 ms (∗ −*p* < 0.05, shown by *red double headed arrows*). GFP and RFFL normalized *I*_to_ recovery curves were fitted with the equation: *I*_to_ = *I*_to,f_ (1 - *exp*(*-Δt/*τ_f_)) + *I*_to,s_ (1 - *exp*(-*Δt*/τ_s_)). The fitting curves are depicted by *thick solid lines* for GFP- (*black*) and RFFL-expressing cells (*red*). The *I*_to,f_ components of the fittings presented by *thin solid lines* and *I*_to,__s_ components are presented by *dashed lines*. The *I*_to,f_ maximal amplitudes in RFFL- *versus* GFP-expressing cells are significantly different, as well as *I*_to,s_ maximal amplitudes (∗∗ −*p* < 0.01). ARbCM, adult rabbit ventricular cardiomyocyte; RFFL, RING finger and FYVE-like domain–containing E3 ubiquitin protein ligase.

To investigate the effect of RFFL on *I*_to_, we transduced ARbCMs with adenovirus encoding GFP or RFFL, and 48 h later, we measured *I*_to_ in the whole-cell configuration. The holding potential was −70 mV and *I*_to_ was evoked by a voltage step to +50 mV. Figure 1, *A* and *B* show a representative experiment with overlapping *I*_to_ traces evoked by a paired pulse protocol with varying interpulse intervals (Δt, see details in the Experimental procedures) in GFP-expressing ARbCMs. Figure 1*C* shows an experiment similar to Figure 1*A*, but with ARbCMs-expressing RFFL. The top inserts in Figure 1, *A* and *C* show two pulses with Δt = 5s, as an example of 1 out of 15 different pulse protocols with various interpulse durations. The first (noninactivated) *I*_to_ peak amplitudes were similar in these two groups: 8.7 ± 1.0 pA/pF in GFP-expressing ARbCMs (n = 12) and 8.3 ± 1.6 pA/pF in RFFL-expressing cells (n = 10).

Traces of *I*_to_ recovery from inactivation after the first pulse are shown in Figure 1, *A* and *C*. Yet, the datasets analyzed with a mixed effects model test did not show any significant difference between GFP- and RFFL-expressing cells, due to the large variation of *I*_to_ amplitudes between individual cells.

The fast inactivation time constants of *I*_to_ for GFP- and RFFL-expressing cells were 22 ± 1.5 and 19 ± 0.8 ms, correspondingly (*p* = 0.26), while slow inactivation constants were 180 ± 16 and 218 ± 58 ms, correspondingly (*p* = 0.32). Thus, we found no significant difference neither in the fast *I*_to_ inactivation kinetics nor in the slow *I*_to_ inactivation kinetics between GFP- and RFFL-expressing myocytes.

Using normalized amplitude data in which the second *I*_to_ peak amplitude was divided by the corresponding first peak amplitude in each paired pulse recording (Fig. 1*D*), we found a significant difference between the *I*_to_ recovery curves of GFP- and RFFL-expressing cells by utilizing a mixed effects model test (*p* < 0.05). In addition, at both 400 ms and 600 ms interpulse intervals the *I*_to_ values in RFFL- and GFP-expressing cells are significantly different (*p* < 0.05, Student’s *t* test). Thus, RFFL suppresses *I*_to_ recovery at 0.4 and 0.6 s interpulse intervals (red asterisks in Fig. 1*D*).

To clarify the origin of the difference between the *I*_to_ recovery curves, we fit these two curves with a sum of two exponential recovery functions, described by the following equation: *I*_to_ = *I*_to,f_ (1 - *exp*(*-Δt/*τ_f_)) + *I*_to,s_ (1 - *exp*(-*Δt*/τ_s_)). The best fits of the curves gave us recovery time constants for *I*_to,f_ components (τ_f_) equal to 50 ± 10 ms and 18 ± 8 ms, but they were not statistically different (*p* > 0.05) due to large variation of measured *I*_to,s_ because of relatively large partially uncompensated capacitive transients in these short recovery time intervals (<100 ms). At the same time, *I*_to,s_ recovery components (τ_s_) were equal to 2.5 ± 0.3 s and 2.8 ± 0.2 s for GFP- and RFFL-expressing cells, respectively (Fig. 1*D*). Thus, we did not uncover any significant effects of RFFL on the time constants of *I*_to_ recovery.

However, in control, GFP-expressing cells, the *I*_to,f_ maximal amplitude (black thin line level at 30 s in Fig. 1*D*) contributed 35 ± 3% to the total *I*_to_ (black thick line), while *I*_to,s_ (black dashed line) provided the remaining 65 ± 3%. Whereas, in RFFL-expressing cells, the *I*_to,f_ amplitude (red thin line) contributed only 21 ± 2%, which was significantly less than in GFP-expressing cells (*p* < 0.01). Correspondingly, the slow component in RFFL-expressing cells (red dashed line) provided 79 ± 2% to the total current (thick red line), which was significantly larger than in GFP-expressing cells (*p* < 0.01). Thus, these results imply that RFFL overexpression in ARbCMs has opposite effects on the expression of Kv4.3-encoded *I*_to,f_, and Kv1.4-encoded *I*_to,s_.

### RFFL downregulates *I*_to,f_ and Kv4.3 expression in a RING domain–dependent manner

We used HEK293A cells to test whether we could use this model to recapitulate the effect of RFFL on *I*_to_ seen in ARbCMs. For the electrophysiological experiments, HEK cells were stably transfected with an expression plasmid encoding Kv4.3, the main pore-forming subunit for *I*_to,f_ in the hearts of rabbits, and transiently cotransfected with plasmids encoding enhanced GFP, RFFL with GFP (RFFL) or RING domain deleted RFFL with GFP (RFFL-ΔRING) with or without the Kv4.3 accessory subunit Kv channel-interacting protein 2 (KChIP2) with DsRed (see Experimental procedures for details).

**Figure 2:**
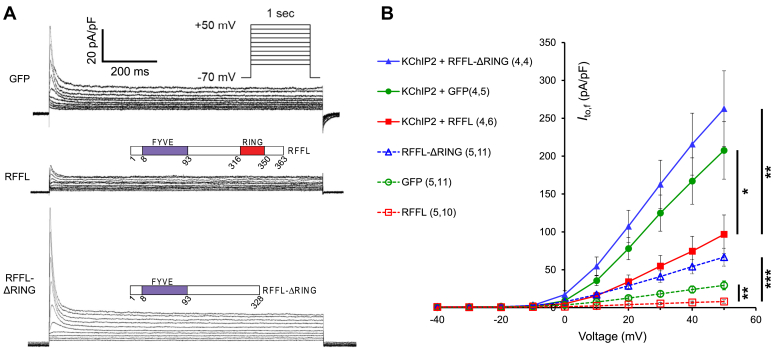
**RFFL downregulates *I***_**to,f**_**in HEK cells stably expressing Kv4.3 in a RING domain–dependent manner independently of KChIP2.***A*, representative traces of *I*_to,f_ in Kv4.3-expressing HEK cells transiently expressing GFP (control), RFFL and GFP, or RFFL-ΔRING and GFP. *Top inset* shows voltage protocol. *Bottom inset* shows *cartoon* of the constructs. *B*, cumulative I-V curves of *I*_to,f_ in Kv4.3-expressing HEK cells transiently expressing GFP (control, *green lines and symbols*), RFFL and GFP (*red*), RFFL-ΔRING and GFP (*blue*), in the presence of KChIP2 and DsRed (*solid lines and symbols*) and the absence of KChIP2 but with DsRed (*dashed lines and open symbols*). The first number in *parentheses* in the legend corresponds to the number of experiments (N) and the second number to the total number of the cells for each condition (n). A mixed effects model for repeated measures data test indicates statistically significant differences between the I-V curves, where ∗ corresponds to *p* < 0.05, ∗∗ −*p* < 0.01, ∗∗∗ −*p* < 0.001. ARbCM, adult rabbit ventricular cardiomyocyte; HEK, human embryonic kidney; KChIP2, Kv channel-interacting protein 2; RFFL, RING finger and FYVE-like domain–containing E3 ubiquitin protein ligase.

We found that RFFL inhibited *I*_to,f_ in a RING domain–dependent manner in the absence of KChIP2 (*p* < 0.01) and in the presence of KChIP2 (*p* < 0.05, Fig. 2). Similar to previous studies in HEK cells (15, 16), we noticed a robust, that is, 7-fold increase in *I*_to,f_ caused by coexpressed KChIP2 alone (*p* < 0.001, solid and dashed green lines in Fig. 2*B*). In the presence of KChIP2, RFFL coexpression caused a 2-fold decrease in *I*_to,f_ relative to GFP-only coexpressing cells (*p* < 0.05). This decrease is similar to the 1.7-fold decrease of the fast-recovering *I*_to_ component in ARbCMs caused by RFFL transduction (Fig. 1*D*).

To clarify whether the effects of RFFL on *I*_to,f_ are due to changes in Kv4.3 and/or KChIP2 trafficking or because of modifications of its intrinsic properties, we analyzed the inactivation kinetics of *I*_to,f_ measured in HEK cells expressing Kv4.3 and KChIP2. Kv4.3 *I*_to,f_ has fast and slow inactivating components. These components in GFP-expressing cells had 16.9 ± 0.7 and 77 ± 15 ms inactivation time constants. While in RFFL-expressing cells these time constants were 15.0 ± 1.3 and 87 ± 23 ms. The ratio of the amplitudes of the fast to slow inactivating components of *I*_to,f_ (*i.e.*, amplitude of *I*_to,f,fast-inactivating_/amplitude of *I*_to,f,slow-inactivating_) in GFP- and RFFL-expressing cells were 14.0 ± 2.2 and 8.9 ± 3.4, respectively. Thus, there were no significant differences in Kv4.3 produced *I*_to,f_ kinetics between GFP- and RFFL-expressing cells. This implies that RFFL does not modify the kinetics of inactivation of Kv4.3 produced *I*_to,f_.

**Figure 3:**
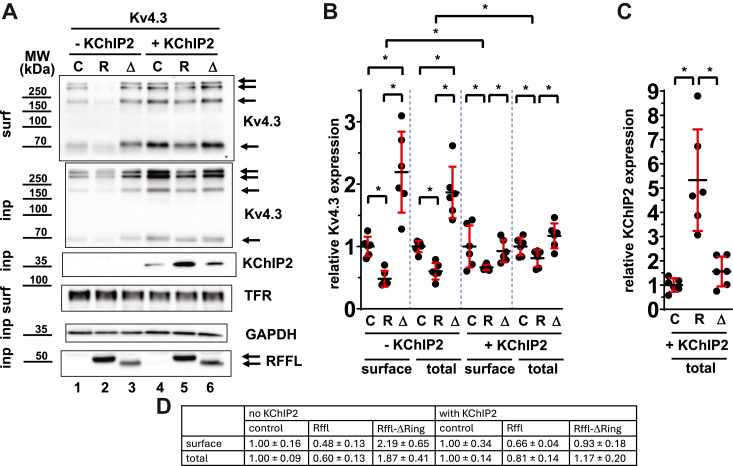
**RFFL downregulates Kv4.3 expression in HEK cells in a RING domain–dependent manner.***A*, protein surface expression of HEK cells, transiently transfected with plasmids for Kv4.3, control (*C*), RFFL (R), RFFL-ΔRING deletion (Δ), and/or KChIP2 was determined by cell surface biotinylation. Surface and input levels of Kv4.3, transferrin receptor (TFR), GAPDH, Flag-RFFL (Flag-RFFL-ΔRING), and KChIP2 are shown. *B*, relative protein expression levels (±SD) of surface (surf) and input (inp) Kv4.3 levels normalized to GAPDH (input) or TFR expression (surface) (N = 4, ∗ −*p* < 0.05). *C*, relative total KChIP2 levels normalized to GAPDH obtained from Kv4.3 surface biotinylation experiments. (N = 6; ∗ −*p* < 0.05). *D*, data table for the relative Kv4.3 expression levels shown in *panel B*. HEK, human embryonic kidney; KChIP2, Kv channel-interacting protein 2; RFFL, RING finger and FYVE-like domain–containing E3 ubiquitin protein ligase.

Next, we wanted to assess whether Kv4.3 levels on the membrane were indeed impacted by RFFL ubiquitination activity. To this end, we transiently expressed Kv4.3, RFFL, its RING deletion (RFFL-ΔRING), which is devoid of ubiquitination activity, or a control plasmid in HEK cells for 48 h. Surface biotinylation assays were conducted to look at the respective surface and total levels of Kv4.3. Cell surface and/or input levels of monomeric Kv4.3, Flag-tagged RFFL, transferrin receptor, and GAPDH are shown in Figure 3*A* (lanes 1–3). As anticipated, we noticed a robust downregulation of cell membrane (−52%) and total Kv4.3 (−40%) in the presence of RFFL (Fig. 3, *B* and *D*). This effect was dependent on the functional Ub ligase domain, as coexpression of RFFL-ΔRING not only prevented the RFFL-dependent downregulation of Kv4.3 levels but rather had the opposite effect, resulting in an approximately 2-fold increase in total and membrane levels of Kv4.3 as compared to control.

As KChIP2 is an important accessory subunit for Kv4.3 to fully reproduce *I*_to,f_, we performed aforementioned surface biotinylation experiments in the presence of coexpressed KChIP2. As shown in Figure 3*A* (lanes 4–6), RFFL still lowered both cell membrane (−34%) and total Kv4.3 levels (−19%), albeit this effect was significantly mitigated compared to the negative effect of RFFL on Kv4.3 in the absence of KChIP2 (Fig. 3, *B* and *D*). Coexpressed KChIP2 also abolished the positive effect of the RING deletion on total and surface Kv4.3 levels. We also noticed that KChIP2 levels, in the presence of Kv4.3, were approximately 5-fold increased by RFFL and that this significant effect relied on the intact RING domain of RFFL (Fig. 3*C*). The biochemical data somewhat supports earlier findings that KChIP2 protects Kv4.3 from degradation in the ER and promotes its forward trafficking (15) and implies a role for overexpressed RFFL in an ER-dependent degradation of Kv4.3.

It should be noted that in the Western blot analysis, we measure changes from thousands of cells, while in electrophysiology we measure the currents only from dozens of “healthy looking” cells, and we cannot expect linear correlation between these the data obtained by different methods. However, the expression levels of surface Kv4.3 at all studied conditions (Fig. 3*A*) changed in the same directions as the corresponding *I*_to,f_ amplitudes (Fig. 2*B*).

### RFFL upregulates *I*_to,s_ and Kv1.4 expression in a RING domain–dependent manner

**Figure 4:**
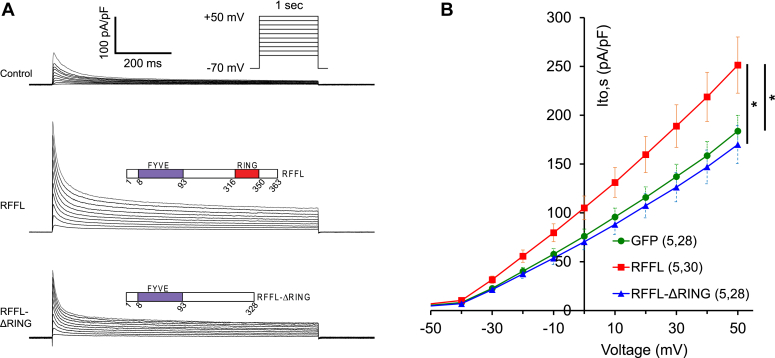
**RFFL upregulates *I***_**to,s**_**in HEK cells stably expressing Kv1.4 in a RING domain–dependent manner.***A*, representative traces of *I*_to,s_ in Kv1.4-expressing HEK cells transiently expressing GFP (control), RFFL and GFP, or RFFL-ΔRING and GFP. *B*, cumulative I-V curves of *I*_to,s_ in HEK cells expressing GFP (*green*), RFFL and GFP (*red*), or RFFL-ΔRING and GFP (*blue*). A mixed effects model for repeated measures data test indicates statistically significant differences between the I-V curves, where ∗ corresponds to *p* < 0.05. HEK, human embryonic kidney; RFFL, RING finger and FYVE-like domain–containing E3 ubiquitin protein ligase.

To investigate the effects of RFFL on *I*_to,s_, HEK cells were stably transfected with an expression plasmid for Kv1.4 and transiently cotransfected with plasmids encoding GFP (control), RFFL, or RFFL-ΔRING mutant. The amplitude of *I*_to,s_ in HEK cells expressing RFFL was significantly larger than in GFP-expressing cells (*p* < 0.05, Fig. 4). This RFFL effect required an intact RING domain. The amplitude of *I*_to,s_ in RFFL-ΔRING–expressing cells was significantly smaller than in the cells expressing RFFL (*p* < 0.05, Fig. 4). The difference between the *I*_to,s_ amplitude in RFFL-ΔRING–expressing cells and GFP-expressing cells was not significant (*p* > 0.05, Fig. 4).

To determine whether increased RFFL changes Kv1.4 trafficking or modifies its channel properties, we analyzed inactivation kinetics of *I*_to,s_ measured in HEK cells expressing Kv1.4. The fast and slow inactivation time constants in GFP-expressing cells were 19.2 ± 1.2 and 226 ± 10 ms, respectively. Whereas in RFFL-expressing cells, the constants were 18.5 ± 0.8 and 211 ± 11 ms. The ratio of the amplitudes of the fast to slow inactivating components of *I*_to,s_ in GFP- and RFFL-expressing cells were 5.4 ± 0.3 and 5.6 ± 0.3, correspondingly. Therefore, similar to Kv4.3, RFFL did not modify the Kv1.4-encoded *I*_to,s_ kinetics of inactivation. This suggests that RFFL primarily affects Kv1.4 trafficking, rather than *I*_to,s_ kinetics.

**Figure 5:**
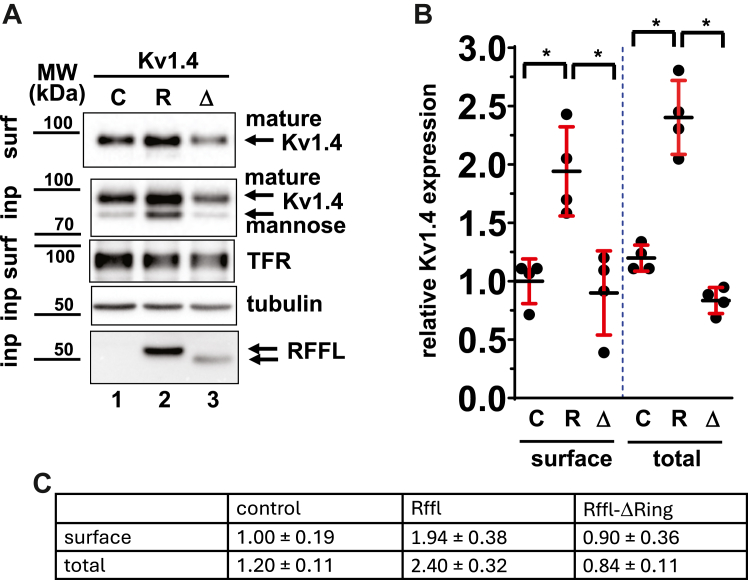
**Cell surface expression of Kv1.4 in HEK cells is upregulated by RFFL in a RING domain–dependent manner.** Protein surface expression of HEK cells transiently transfected with plasmids for Kv1.4, control (C), RFFL (R), or RFFL-ΔRING mutant (Δ) was determined by cell surface biotinylation. *A*, representative Western blots depicting cell surface (surf) and input (inp) levels of Kv1.4 (mature [sialylated N-glycans] and immature, [high mannose N-glycans] (56), transferrin receptor (TFR), tubulin, and Flag-RFFL. *B*, relative protein expression levels (±SD) of Kv1.4 normalized to tubulin (total) or TFR expression (surface) (N = 4, ∗ −*p* < 0.05). Cell surface and input levels of Kv1.4 (mature and immature channels), transferrin receptor (TFR), tubulin, and Flag-RFFL are shown. (N = 4, ∗ −*p* < 0.05). *C*, data table for the relative Kv1.4 expression levels shown in *panel B*. HEK, human embryonic kidney; RFFL, RING finger and FYVE-like domain–containing E3 ubiquitin protein ligase.

To that end, surface biotinylation experiments were conducted to study the effect of RFFL on surface and total levels of coexpressed Kv1.4. HEK cells were transiently transfected with plasmids encoding Kv1.4 and RFFL, RFFL-ΔRING, or control plasmid for 48 h. Cell surface and/or input levels of monomeric Kv1.4, transferrin receptor, RFFL, RFFL-ΔRING, and tubulin are shown in Figure 5*A*. As expected, the biotin fraction representing the surface proteins revealed mostly mature-type Kv1.4 glycoprotein (*trans-*Golgi glycosylated Kv1.4; Fig. 5*A*; top row) (17), while the input fraction presents a mixture of high mannose-type (mainly found in the ER) and mature-type Kv1.4 glycoprotein (Fig. 5*A*; second row from top). Cumulative data showed that RFFL overexpression significantly increased total (+94%) and surface expression (+140%) of both glycosylated forms of Kv1.4 (*p* < 0.05) (Fig. 5, *B* and *C*). The RFFL-dependent increase in the expression of the high-mannose ER-resident form of Kv1.4 would also suggest that RFFL likely exerts its effect on Kv1.4 channels on the membrane of the ER. Deletion of the RING domain of RFFL, however, fully eliminated the upregulation of Kv1.4 at both the surface and total levels (Fig. 5*B*). Thus, the RING-dependent positive effect of RFFL on Kv1.4 surface expression is in agreement with the corresponding change in *I*_to,s_ amplitude (Fig. 4).

### Computer modeling of RFFL influence on rabbit action potential

Previously, we have shown that overexpressed RFFL downregulates *I*_Kr_ and hERG protein in adult rabbit cardiomyocytes and HEK293A cells (12). Here, we show that RFFL also downregulates *I*_to,f_ (Figs. 1*D* and 2*B*) and Kv4.3 (Fig. 3*B*) but upregulates *I*_to,s_ (Figs. 1*D* and 4*B*) and Kv1.4 (Fig. 5). To determine the effect of RFFL on the cellular excitability and APD at 90% repolarization level (APD90), we conducted electrophysiological experiments with adenovirally transduced ARbCMs. Yet, a large variation of APD90 values in these remodeled cultured cells did not allow us to determine a statistically significant difference in APD90 between the cells expressing GFP and RFFL (data not shown).

**Figure 6:**
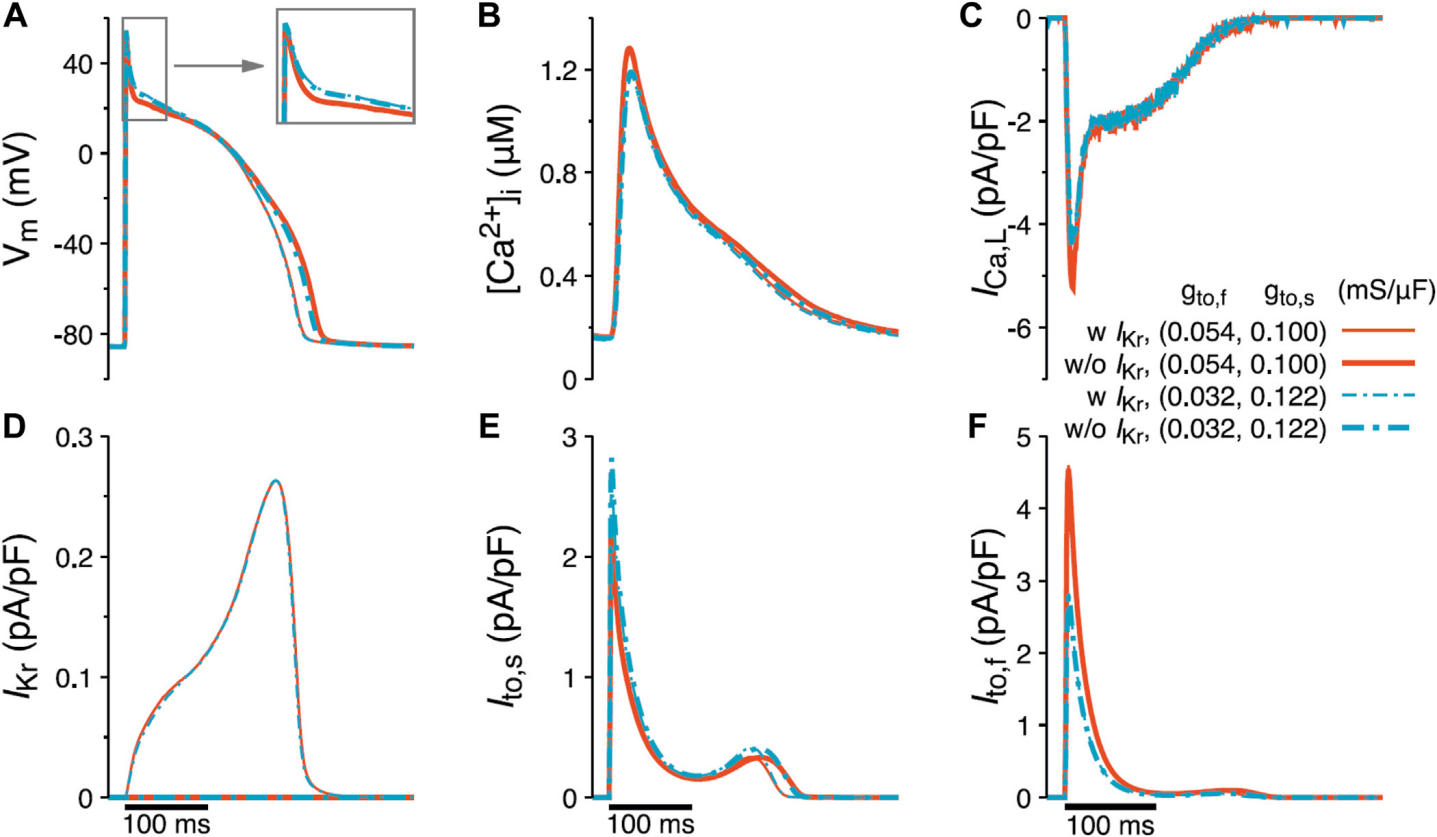
**Computer modeling shows that the change of *I***_**to,f**_**and *I***_**to,s**_**associated with RFFL overexpression affects action potential duration for both normal and fully suppressed *I***_**Kr**_**.***A*, simulated AP traces (400 ms cycle length) in a control cell (*thin red line*) and a cell under different *I*_Kr_ and/or *I*_to_ conditions associated with RFFL overexpression, which suppresses *I*_Kr_, downregulates g_to,f_, and upregulates g_to,s_. The *thick red line* represents the AP with *I*_Kr_ suppressed only. The *dash-dotted thin blue line* shows the effect of g_to,f_ downregulation and g_to,s_ upregulation only. The *dash-dotted thick blue line* exhibits the combination effect on AP by suppressing *I*_Kr_, decreasing g_to,f_, and increasing g_to,s_, that is, the consequence of overexpressing RFFL. During an AP shown in (*A*), the time dependences of [Ca^2+^]_i_, *I*_Ca,L_, *I*_Kr_, *I*_to,s_, and *I*_to,f_ are shown in (*B*–*F*), respectively. AP, action potential; RFFL, RING finger and FYVE-like domain–containing E3 ubiquitin protein ligase.

Therefore, we utilized our spatially detailed model for rabbit ventricular myocytes (18) and presented here assessments of RFFL effects on the AP at 2.5 Hz stimulation in four different conditions:

1. control (g_to,f_ = 0.054 mS/μF, g_to,s_ = 0.1 mS/μF, and normal g_Kr_).
2. RFFL caused full suppression of only *I*_Kr_ with normal *I*_to_ (g_to,f_ = 0.054 mS/μF, g_to,s_ = 0.1 mS/μF, and g_Kr_ = 0).
3. RFFL modified *I*_to_ with normal *I*_Kr_ (g_to,f_ = 0.032 mS/μF, g_to,s_ = 0.122 mS/μF, and normal g_Kr_).
4. RFFL modified *I*_to_ and completely inhibited *I*_Kr_ (g_to,f_ = 0.032 mS/μF, g_to,s_ = 0.122 mS/μF, and g_Kr_ = 0), (Fig. 6).

Computer modeling shows that at a stimulation of 2.5 Hz, the combined effect of a decrease in *I*_to,f_ and an increase in *I*_to,s_, mimicking RFFL overexpression, reduces APD90 only by 3.0 ms and 3.9 ms with normal and completely inhibited *I*_Kr_, respectively (Fig. 6*A*). The decrease in *I*_to,f_ (Fig. 6*F*) led to a smaller AP notch and thicker AP overshoot (Fig. 6*A* insert), while the increase in *I*_to,s_ was the major contributor to APD90 shortening both with normal and completely inhibited *I*_Kr_ (Fig. 6, *A*, *D*, and E). However, the major effect of RFFL on APD90 is due to its suppression of *I*_Kr_ (Fig. 6, *A* and *D*), which leads to an increase of 20.6 ms in APD90 when *I*_to,f_ and *I*_to,s_ are unaffected (Fig. 6*A*). With the changes in *I*_to,f_ and *I*_to,s_ included (Fig. 6, *E* and *F*), the increase in APD90 due to *I*_Kr_ suppression is slightly reduced to 19.7 ms (dot-dashed *versus* solid lines in Fig. 6*A*). Furthermore, the overexpression of RFFL also affects Ca^2+^ cycling. The more positive notch when *I*_to,f_ and *I*_to,s_ are affected results in a decreased peak of the L-type Ca^2+^ current (I_Ca_) (Fig. 6*C*) and, consequently, a reduced peak of intracellular Ca^2+^ concentration (Fig. 6*B*). Additionally, the reduced prolongation of APD90 leads to a slight decrease in intracellular Ca^2+^ concentration during the decay phase (Fig. 6*B*). In other words, the effect of RFFL overexpression induced changes to APD90 *via I*_to,f_ and *I*_to,s_, and the effect of the notch potential on I_Ca_ partially counteracts the impact of *I*_Kr_ suppression by RFFL overexpression.

The APD90 prolongation of 20 ms due to the full *I*_Kr_ suppression may seem inconsistent with the much more significant prolongations observed in existing experimental LQT2 rabbit models (19). However, it is notable that this LQT2 rabbit model involves an additional 30% inhibition of *I*_Ks_, a longer pacing cycle length, and the presence of isoproterenol. As shown in Table S3 produced by our computational rabbit model, an additional 30% inhibition of *I*_Ks_ increases the APD90 from 217 ms to 266 ms, that is, by 49 ms. Under this condition, RFFL-induced *I*_to_ changes reduce APD90 by 8 ms (from 266 ms to 258 ms). With the stimulation frequency decreased from 2.5 Hz to 0.25 Hz, which is close to the experimental frequency in Ref. (19), the APD90 increases from 284 ms to 349 ms, that is., by 65 ms, while RFFL-caused *I*_to_ changes have no noticeable effect on APD90. Furthermore, in the presence of isoproterenol and at the cycle length of 4 s, the full *I*_Kr_ blockade and 30% suppression of *I*_Ks_ prolong the APD90 by 94 ms (from 322 ms to 416 ms), and the RFFL-caused *I*_to_ changes reduce the APD90 by 13 ms (from 416 ms to 403 ms). It is notable that this APD90 prolongation by 94 ms quantitatively agrees with the experimental observations of the LQT2 rabbit model (20, 21). In addition to the effect of *I*_Kr_ suppression, these results also demonstrate that the effect of the RFFL-produced *I*_to_ modifications on APD90 depends on the background conditions and other channels.

## Discussion

Introgression of a congenic strain of Dahl salt-sensitive rats with genomic segments from the normotensive Lewis rat allowed mapping of a quantitative trait locus for hypertension, short QT interval, and cardiac hypertrophy to a 42.5-kb region that contains only the *RFFL* gene (14). As the transient outward potassium current *I*_to_ is the major repolarizing current in rat ventricular myocytes (22, 23), we set out to look for any functional interaction between RFFL and *I*_to_ using cultured adult rabbit cardiomyocytes. However, dedifferentiation of cultured rabbit cardiomyocytes (24, 25, 26) may confound any findings. To minimize dedifferentiation in this study, we cultured adult rabbit cardiomyocytes no more than 48 h and used only healthy looking, rod-shaped, striated cardiomyocytes for patch-clamp experiments. We also added 0.5 μM cytochalasin D to the medium to preserve the function (*e.g.*, APs and calcium transients) and morphology (rod-shape and T-tubular structure) of cardiomyocytes as published elsewhere (27). In the past, we used 0.5 μM cytochalasin D to prevent dedifferentiation in 3-week-old adult rabbit cardiomyocytes cultured for 48 h (28). Although some functional parameters, such as resting potential and *I*_Na_, did not change after a 48-h culture of cardiomyocytes, others did (*e.g.*, APD prolongation, increase in *I*_Ca_, and decrease in *I*_K1_ or *I*_Kr_). Importantly, *I*_to_ did not change in these cardiomyocytes cultured for 48 h. Despite the efforts to minimize cardiomyocyte remodeling, we acknowledge the possibility that exogenous RFFL, a RING finger Ub ligase with a plethora of target molecules, may impact the dedifferentiation process in cultured cells, which in turn may explain the reciprocal changes of *I*_to,f_ and *I*_to,s_. Yet, we do believe that there is a direct functional interaction between RFFL and *I*_to_ in mammalian cardiomyocytes as implied by the aforementioned study of a congenic rat strain (14). Importantly, we were able to reproduce the yin-yang effect of RFFL on *I*_to,f_ and *I*_to,s_ in heterologous HEK293A cells.

Our *I*_to_ recovery time constants in GFP-expressing cultured ARbCMs (Fig. 1*D*) are similar to previously published parameters obtained in acutely isolated ARbCMs, τ_f_ = 22 ± 1 ms and τ_s_ = 2.78 ± 0.06 s (29). However, the relative contributions of the fast and slow components to the total *I*_to_, 16 ± 2% and 83 ± 6%, respectively (29), differ from our data. Here, we show that the relative contributions are 35 ± 3% and 65 ± 3%, respectively (Fig. 1*D*). Therefore, in the experiments with cultured primary cardiac myocytes, we alternated patch-clamp recordings from RFFL- and GFP-expressing cells to equalize the culturing time between the two types of cells before the measurements.

It is known that the manifestation of *I*_to,f_ and *I*_to,s_ is not homogeneous in the heart and it exhibits expression gradients of these two *I*_to_ currents across the free wall, as well as changes in expression of auxiliary subunits and phosphorylation by CaMKII (30, 31). Our study shows increased complexity of *I*_to_ regulation because of a yin and yang effect of RFFL on the fast and slow *I*_to_ components. While RFFL has effects on both fast and slow *I*_to_ components (Fig. 1*D*), the maximal amplitude of total *I*_to_ remains practically unchanged. Since the major action of RFFL on the repolarization during the AP is the suppression of *I*_Kr_ (12), the effect of RFFL reported in this study may be considered as a combination of LQT2 syndrome (lack of *I*_Kr_) and a modulation of *I*_to_ expression. Incidentally, in electrophysiological terms, this situation is similar to the effects of *I*_to_ in the LQT1 rabbit heart, where the *I*_Ks_ current is suppressed, while *I*_to_ kinetics variation leads to polymorphic ventricular tachycardia under isoproterenol (32). When we introduced changes in *I*_Kr_ and *I*_to_ corresponding to RFFL overexpression using our spatially detailed model for rabbit ventricular myocytes (18), the APD90 was prolonged by 20.6 ms (*i.e.*, 10%) due to the *I*_Kr_ suppression alone. On top of that, the effects of *I*_to_ led to a 3.9 ms (*i.e.*, 2%) decrease in APD90 (Fig. 6). This result demonstrates that the effects of RFFL on APD90 *via* the changes in *I*_Kr_ and *I*_to_ have opposite trends. The dominant contribution of *I*_to_ to the small APD90 shortening is the increase from 65% to 79% of *I*_to,s_ relative to total *I*_to_ in ARbCMs (Figs. 1*D*, and 6, *A* and *E*), which correlates with an increase in Kv1.4 expression in HEK cells (Figs. 4 and 5). In contrast, the RFFL evoked reduction of *I*_to,f_ in ARbCMs (Figs. 1*D* and 6*F*) corroborated the decrease in Kv4.3 expression in HEK cells (Fig. 3) and resulted in a small reduction of the AP notch and a thicker AP overshoot in the computer model (Fig. 6*A*). Thus, RFFL overexpression leads to the reduction of *I*_Kr_ and increase in *I*_to,s_ that have opposing effects on APD90, while the reduction of *I*_to,f_ slightly broadens the AP peak. The effect of RFFL on the AP will likely be more complicated under isoproterenol (32), which requires additional studies.

The nature of the opposing effects of RFFL on the fast and slow components of *I*_to_ and on expression of Kv4.3 and Kv1.4 is yet to be determined. The complexity of ubiquitination outcomes is the result of different types of ubiquitinations, such as monoubiquitination, multimonoubiquitination, homotypic ubiquitination, heterotypic ubiquitination, modified ubiquitination, etc., which lead to different consequences (33). In addition, there is the Ub-proteasome pathway–mediated Kv4.3 degradation, which apparently does not involve RFFL (34). There are also other enzymes that affect trafficking of the channel proteins underlying *I*_to_, which potentially might also be affected by RFFL (23, 35). In contrast, another ring finger protein E3 ligase, RNF207, increases expression of hERG (36), while on the other hand it exacerbates pathological cardiac hypertrophy *via* posttranslational modification of TAB1 (37).

A further complication of the RFFL effect on *I*_to_ is that native Kv4.x channels need to assemble with KChIPs to fully reproduce the *I*_to_ current (38, 39). KChIP2 proteins are expressed in human hearts, and KChIP2 overexpression in Kv4.3-transfected HEK293A cells increased *I*_to_ and slowed the *I*_to_ decay rate (40), which increases the repolarization reserve. KChIP2 also affects the trafficking of Kv4.2 by masking an N-terminal Kv4.2 hydrophobic domain (41). Since we have shown that RFFL overexpression increased the total expression of KChIP2 (Fig. 3*C*), and since KChIP2 overexpression increases expression of Kv4.3 (Fig. 3*C*) and *I*_to_ in the control (Fig. 2*B*), then RFFL has two opposing effects on *I*_to,f_. On the one hand, RFFL overexpression increases *I*_to,f_ by increasing KChIP2 (Fig. 3*C*) and consequently Kv4.3 (Fig. 3*A*, compare two “R” lanes) expressions, but on the other hand, RFFL reduces total and surface Kv4.3 expression (Fig. 3*A*, compare “C” and “R” lanes). Given that *I*_to,f_ in rabbits was suppressed by RFFL overexpression, we conclude that the inhibiting effect of RFFL overexpression on Kv4.3 and *I*_to,f_ directly dominates over the indirect upregulation of Kv4.3 due to upregulation of KChIP2 in rabbit myocytes.

To our knowledge, this is the first report directly linking ubiquitination to *I*_to_ currents encoded by Kv1.4 and Kv4.3. Surface biotinylation experiments and patch clamp studies (Figure 2, Figure 3, Figure 4, Figure 5) suggest that RFFL expression regulates surface expression of both channels in a reciprocal manner. Importantly, these effects of RFFL are dependent on a functional RING domain. We used Rapid UBIquitination sequence detection, a sequence-based predictor for ubiquitinated lysines (42), to look for potential ubiquitination sites in both channels. Not surprisingly, ubiquitination sites were predicted for human Kv1.4 (lysine 268) and Kv4.3 (lysine 71). Both sites are conserved in rabbit. Next, we used UbiBrowser (43), a bioinformatics platform that predicts Ub ligase substrate interactions. Although UbiBrowser did not predict any interactions between RFFL and Kv1.4 or Kv4.3, it identified potential interactions between RFFL and three (co) chaperones (three out of a total of 117 predicted RFFL substrates) involved in protein quality control: HSP5A (BiP), a member of the Hsp70 family involved in the folding of proteins in the ER, BAG3, and BAG6, which are both cochaperones of Hsp70 and are also found localized to the ER (44, 45, 46). The implication of a functional interaction between RFFL and (co) chaperones is particularly interesting given our published results showing that RING finger protein RNF207, another Ub ligase involved in cardiac excitation, regulates hERG trafficking in a heat shock–dependent manner, likely *via* interaction with cytosolic chaperones such as Hsp70 and Hsp40 on the ER, to assist synthesis, folding, and/or ER export of hERG (36). One can envisage that RFFL ubiquitinates various (co) chaperones in a similar way as it monoubiquitinates effectors of Rab11, a crucial regulator of intracellular membrane trafficking such as recycling of endocytosed membrane proteins (6, 47). Indeed, degradation-independent monoubiquitination of Hsp70 and Hsc70 was previously reported for the RING finger Ub ligase parkin (48), though functional consequences were not seen. Monoubiquitination of (co) chaperones may affect their catalytic activities with respect to folding, assembly, forward trafficking, and degradation of client proteins. In the case of Kv1.4, monoubiquitination of (co) chaperones by RFFL may result in increased surface expression of the channel. Alternatively, RFFL may directly ubiquitinate Kv1.4 to increase its stability. However, unlike for hERG (12), we were not able detect any RFFL-dependent ubiquitination of Kv1.4 using immunoprecipitations. It is also plausible that the effect of RFFL may rather be mediated through an indirect effect, for example, by inhibiting a negative regulator of Kv1.4. We previously showed that RFFL-mediated polyubiquitination of hERG channel resulted in its proteasomal degradation, which relied on a functional ER-associated degradation pathway in 293A cells (12). Likely, the same molecular mechanism underlies the degradation of Kv4.3 by RFFL in the absence of KChIP2. As KChIP proteins are known to stabilize Kv4 α subunit channel complexes and promote channel assembly and/or trafficking to the membrane (49, 50, 51), we speculate that KChIP2 substantially protects Kv4.3 from RFFL-mediated degradation. The observed positive effect of RFFL harboring a deletion of the RING domain on Kv4.3 surface expression could be due to the formation of inactive complexes that can no longer promote Kv4.3 ubiquitination/degradation. A possible candidate inhibited by the RING deletion of RFFL could well be CHIP, a U-box–dependent Ub ligase, and cochaperone important for the balance between protein degradation and folding (52). Functional interactions between RFFL and CHIP may exist on the ER as both molecules share overlapping functions in the peripheral protein quality control of misfolded CFTR (9, 53). For example, CHIP and parkin are known to functionally interact during the degradation of unfolded PaeI receptor in dopaminergic neurons (54). Further research is warranted to delineate the molecular mechanisms underlying the reciprocal regulation of both *I*_to_ channels by (in) direct ubiquitination through RFFL. Of note, recent experiments showed that brefeldin-A inhibited transport of Kv1.4 from the ER to the Golgi complex (data not shown).

In summary, our data suggest that channel stability (Kv4.3) and forward trafficking to the surface (Kv1.4) are regulated by RFFL-dependent ubiquitination events on the ER. These events result in reciprocal changes in surface expression of both channels underlying *I*_to,f_ and *I*_to,s_.

## Experimental procedures

*DNA*-*I*_to_ channel expression plasmids used in this study were generously provided by Dr Jeanne Nerbonne (Washington State University): pCMV-Script-mKChIP2 (murine KChIP2 clone ligated into Xho I/Kpn I sites of pCMV-Script), pCMV-Script-hKv1.4 (human Kv1.4 clone ligated to Xho I/EcoR I sites of pCMV-Script), and pGFP-Ire-hKv4.3L (human Kv4.3 clone encoding the long 650-aa isoform ligated to Sac II/SpeI sites of pGFP Ire). To achieve higher expression levels of Kv4.3, its ORF was ligated into pcDNA3 using Hind III/Not I sites (pcDNA3-Kv4.3L). Expression plasmids for Flag-tagged human RFFL (pFLAG-CMV2-CARP2; Addgene ID 16013) was purchased from Addgene. The deletion of the RING (ΔRING) domain was generated by site-directed mutagenesis to obtain pFLAG-CMV2-RFFL-ΔRING (36). To allow coexpression of RFFL and its RING domain deletion with enhanced green fluorescent protein (EGFP), ORFs of RFFL (or RFFL-ΔRING) and EGFP were cloned downstream the immediate/early promoter enhancer of cytomegalovirus (CMV) and phosphoglycerate kinase (PGK) promoters of the dual expression plasmid pSF-CMV-PGK (MilliporeSigma) using suitable restriction sites (pSF-CMV-RFFL-PGK-EGFP and pSF-CMV-RFFL-ΔRING-PGK-EGFP). To obtain the control vector pSF-CMV-PGK-EGFP, EGFP was cloned downstream of the PGK promoter. Similarly, PCR-amplified c-myc-tagged mKChIP2 and DsRed or DsRed only were cloned into pSF (pSF-CMV-mKChIP2-PGK-DsRed and pSF-CMV-PGK-DsRed). Adenovirus-expressing Flag-tagged RFFL or GFP was prepared using the Gateway cloning system (Thermo Fisher Scientific) as described previously (Ad-RFFL and Ad-GFP) (36).

### Preparation of rabbit cardiomyocytes

All animal experiments and procedures were approved by the Rhode Island Hospital Institutional Animal Care and Use Committee (reference numbers: 0188-14 and 5013-17). Septal ARbCMs were isolated from the hearts of 6- to 24-month-old New Zealand White rabbits (both sexes). The filtered cells were maintained in 45 mM KCl, 65 mM potassium glutamate, 3 mM MgSO_4_, 15 mM KH_2_PO_4_, 16 mM taurine, 10 mM Hepes, 0.5 mM EGTA, and 10 mM glucose (pH 7.3) for 1 h. Cells were allowed to sediment for 30 min, and after removal of the supernatant, resuspended in medium 199 (Thermo Fisher Scientific) supplemented with 5% fetal bovine serum (MilliporeSigma), antibiotics, and 0.5 μM cytochalasin D (MilliporeSigma). After plating cells on laminin-coated cover glasses, adenovirus (50 multiplicity of infection) encoding GFP or RFFL was added to the cells. Cells were maintained at 37 °C with 5% CO_2_ and ∼48 h later the cells were used for patch clamp and biochemistry.

### Stable lines of 293A cells expressing human Kv1.4 and Kv4.3

HEK293A cells (Thermo Fisher Scientific) were transfected with pCMV-Script-hKv1.4 or pcDNA3-Kv4.3L. The cells were seeded in 96-wells into Dulbecco's Modified Eagle's Medium containing 900 μg/ml geneticin. Single clones were isolated and expanded (400 μg/ml geneticin). Suitable clones were checked for expression of Kv1.4. and Kv4.3 by Western blotting and patch clamp.

### Transfections

293A cells were cultured in Dulbecco's Modified Eagle's Medium and 10% fetal bovine serum and split at approximately 50% confluency. For subsequent cell surface biotinylation experiments, performed in 60 mm cell culture dishes, we transfected cells with a total of 2500 ng DNA (*e.g.*, 750 ng pcDNA3-Kv4.3L, 750 ng pSF-CMV-mKChIP2-PGK-DsRed, 375 ng pFLAG-CMV2-RFFL, and 625 ng pcDNA3; 750 ng pCMV-Script-hKv1.4, 600 ng pFLAG-CMV2-RFFL, and 1150 ng pcDNA3) using lipofectamine 2000 (Thermo Fisher Scientific). For transient transfections of stable cell lines, performed in 12-wells prior to electrophysiological recordings, we used 440 ng DNA: for example, 80 ng pSF-CMV-RFFL-PGK-EGFP (pSF-CMV-RFFL-ΔRING-PGK-EGFP or pSF-CMV-PGK-EGFP) and 360 ng pcDNA3 for Kv1.4-expressing cells; and 60 ng pSF-CMV-RFFL-PGK-EGFP (pSF-CMV-RFFL-ΔRING-PGK-EGFP or pSF-CMV-PGK-EGFP), 200 ng pSF-CMV-mKChIP2-PGK-DsRed (pSF-CMV-PGK-DsRed), and 180 ng pcDNA3 for Kv4.3-expressing cells). Transfected cells were incubated for approximately 48 h.

### Electrophysiological recordings

All experiments were conducted in the whole-cell configuration at 35 °C to 37 °C with Axopatch-200B, Digidata 1440A, and pClamp 10 software (Molecular Devices, https://support.moleculardevices.com/s/article/Axon-pCLAMP-10-Electrophysiology-Data-Acquisition-Analysis-Software-Download-Page). The seal and the whole cell configuration were obtained in Tyrode solution (in mM): 140 NaCl, 5.4 KCl, 0.33 NaH_2_PO_4_, 1.8 CaCl_2_, 1 MgCl_2_, 10 Hepes, and 5.5 glucose; pH7.4 was adjusted with NaOH. The cell membrane capacitance and series resistance were compensated by about 70%. To minimize *I*_Na_ at to +50 mV depolarizing voltage step, outward *I*_to_ in ARbCMs was measured in low-sodium Tyrode solution (in mM): 100 N-methyl-D-glucamine, 40 NaCl, 5.4 KCl, 0.33 NaH_2_PO_4_, 1 CaCl_2_, 0.2 MgCl_2_, 5 Hepes, and 7.5 D-glucose; pH was adjusted to 7.4 with HCl. To suppress *I*_Ca,L_, *I*_Kr,_ and *I*_Ks_ in ARbCMs, we added 0.2 mM CdCl_2_, 2.5 μM E−4031, and 30 μM chromanol, correspondingly. The pipette resistance was 2 to 4 MΩ when filled with intracellular solution (in mM): 130 KCl, 10 NaCl, 0.36 CaCl_2_, 5 EGTA, 5 Hepes, 5 D-glucose, 5 Mg-ATP, 5 Na_2_-phosphocreatine, 0.25 Na_2_-GTP; pH 7.2 was adjusted with KOH. To evoke *I*_to_ in ARbCMs, we used a double-pulse protocol from the holding potential of −70 mV. Both depolarizing pulses were to +50 mV and 500 ms in duration, while the recovery intervals between these two paired pulses had different durations (in ms): 10, 20, 30, 50, 100, 150, 200, 400, 600, 1000, 2000, 3000, 5000, 10,000, and 15,000. The durations between any two consecutive paired pulse stimulations were 30 s. To measure *I*_to_ in transfected HEK cells, we used Tyrode solution for the bath and the same pipette solution as described above. The holding potential for the HEK cells was −70 mV and every 15 s depolarizing 1000 ms test pulses were applied in 10 mV increments in the range from −40 mV up to +50 mV. The acquisition rate was 10 kHz with a low-pass filter set at 5 kHz. The *I*_to_ amplitude was calculated as the difference between *I*_to_ peak current and the steady-state current at the end of the test pulse.

### Immunoblot analysis

Cell surface biotinylations and immunoblots were essentially carried out as in previous studies (36). However, prompted by a recent study (55), we introduced the following changes to achieve optimal resolution of transmembrane proteins: All samples were mixed with an equal volume of 4x Laemmli sample buffer (62.5 mM Tris–HCl, pH 6.8, 10% glycerol, 1% lithium dodecyl sulfate, and 0.005% Bromophenol Blue) and incubated at room temperature for 15 (protein samples) or 60 min (biotinylated fractions) prior to gel loading. Membranes were incubated with the following antibodies for 2 h at room temperature: mouse anti-GAPDH (Thermo Fisher Scientific; 39-8600; 1:3000); rabbit anti-Kv4.3 (Chemicon; AB5194; 1:1000); mouse anti-Kv1.4 (UC Davis/NIH NeuroMab Facility; AB_2877317; 1:1000); rabbit anti transferrin receptor (Novus Biologicals; NBP1-85741; 1:1000); mouse anti Oct-A (Flag; Santa Cruz Biotechnology; sc-166355; 1:300); mouse anti c-Myc (Santa Cruz Biotechnology; sc-40; 1:300); and mouse anti-tubulin (Cell Signaling Technology; 3873;1:5000). Suitable secondary horseradish peroxidase-conjugated antibodies (Thermo Fisher Scientific) were used at 1:10,000.

### Rabbit ventricular myocyte model

To study the effect of RFFL in myocytes, we used a physiologically detailed rabbit ventricular myocyte model (18) with 2.5 Hz stimulation frequency. The 100th AP after the beginning of the simulation is shown in Figure 6.

Statistical analysis and curve fitting were performed with GraphPad Prism 8 (https://www.graphpad.com/) and OriginPro 2019 (https://www.originlab.com/2019). If there were outliers in a dataset, they were removed for further analysis. Statistical comparison of multipoint curves was performed with a mixed effects model for repeated measures data test. The fitting of the curves with two exponents were performed in OriginPro with the Levenberg Marquardt iteration algorithm. Statistical comparison of two groups was done with Student’s *t* tests (two-tailed). Data were presented as mean ± SEM for electrophysiological recordings and mean ± SD for immunoblots. A difference was considered significant at *p* < 0.05.

### Replicates

Throughout the study, we used biological replicates, that is, different numbers of animals or different frozen HEK cell stocks (as indicated by “N”), and technical replicates (as indicated by “n”).

## Data availability

All data are contained within the manuscript.

## Supporting information

This article contains supporting information.

## Conflict of interest

The authors declare that they have no conflicts of interest with the contents of this article.

## References

[1] Pfeufer A., Sanna S., Arking D.E., Muller M., Gateva V., Fuchsberger C. (2009). Common variants at ten loci modulate the QT interval duration in the QTSCD Study. Nat. Genet..

[2] Newton-Cheh C., Eijgelsheim M., Rice K.M., de Bakker P.I., Yin X., Estrada K. (2009). Common variants at ten loci influence QT interval duration in the QTGEN Study. Nat. Genet..

[3] Arking D.E., Pulit S.L., Crotti L., van der Harst P., Munroe P.B., Koopmann T.T. (2014). Genetic association study of QT interval highlights role for calcium signaling pathways in myocardial repolarization. Nat. Genet..

[4] Metzger M.B., Hristova V.A., Weissman A.M. (2012). HECT and RING finger families of E3 ubiquitin ligases at a glance. J. Cell Sci..

[5] Cai C., Tang Y.D., Zhai J., Zheng C. (2022). The RING finger protein family in health and disease. Signal. Transduct. Target. Ther..

[6] Sakai R., Fukuda R., Unida S., Aki M., Ono Y., Endo A. (2019). The integral function of the endocytic recycling compartment is regulated by RFFL-mediated ubiquitylation of Rab11 effectors. J. Cell Sci..

[7] Cheng X., Waghulde H., Mell B., Smedlund K., Vazquez G., Joe B. (2016). Pleiotropic effect of a high resolution mapped blood pressure QTL on tumorigenesis. PLoS one.

[8] Gan X., Wang J., Wang C., Sommer E., Kozasa T., Srinivasula S. (2012). PRR5L degradation promotes mTORC2-mediated PKC-delta phosphorylation and cell migration downstream of Galpha12. Nat. Cell Biol..

[9] Okiyoneda T., Veit G., Sakai R., Aki M., Fujihara T., Higashi M. (2018). Chaperone-independent peripheral quality control of CFTR by RFFL E3 ligase. Dev. Cell.

[10] Coumailleau F., Das V., Alcover A., Raposo G., Vandormael-Pournin S., Le Bras S. (2004). Over-expression of Rififylin, a new RING finger and FYVE-like domain-containing protein, inhibits recycling from the endocytic recycling compartment. Mol. Biol. Cell.

[11] Gopalakrishnan K., Kumarasamy S., Yan Y., Liu J., Kalinoski A., Kothandapani A. (2012). Increased expression of rififylin in A <330 Kb congenic strain is linked to impaired endosomal recycling in proximal tubules. Front. Genet..

[12] Roder K., Kabakov A., Moshal K.S., Murphy K.R., Xie A., Dudley S. (2019). Trafficking of the human ether-a-go-go-related gene (hERG) potassium channel is regulated by the ubiquitin ligase rififylin (RFFL). J. Biol. Chem..

[13] Santolini M., Romay M.C., Yukhtman C.L., Rau C.D., Ren S., Saucerman J.J. (2018). A personalized, multiomics approach identifies genes involved in cardiac hypertrophy and heart failure. NPJ Syst. Biol. Appl..

[14] Gopalakrishnan K., Morgan E.E., Yerga-Woolwine S., Farms P., Kumarasamy S., Kalinoski A. (2011). Augmented rififylin is a risk factor linked to aberrant cardiomyocyte function, short-QT interval and hypertension. Hypertension.

[15] Wang N., Dries E., Fowler E.D., Harmer S.C., Hancox J.C., Cannell M.B. (2022). Inducing Ito,f and phase 1 repolarization of the cardiac action potential with a Kv4.3/KChIP2.1 bicistronic transgene. J. Mol. Cell. Cardiol..

[16] Lainez S., Doray A., Hancox J.C., Cannell M.B. (2018). Regulation of Kv4.3 and hERG potassium channels by KChIP2 isoforms and DPP6 and response to the dual K(+) channel activator NS3623. Biochem. Pharmacol..

[17] Watanabe I., Zhu J., Recio-Pinto E., Thornhill W.B. (2004). Glycosylation affects the protein stability and cell surface expression of Kv1.4 but Not Kv1.1 potassium channels. A pore region determinant dictates the effect of glycosylation on trafficking. J. Biol. Chem..

[18] Moshal K.S., Roder K., Kabakov A.Y., Werdich A.A., Chiang D.Y., Turan N.N. (2019). LITAF (Lipopolysaccharide-Induced tumor necrosis factor) regulates cardiac L-type calcium channels by modulating NEDD (neural precursor cell expressed developmentally downregulated protein) 4-1 ubiquitin ligase. Circ. Genom. Precis Med..

[19] Brunner M., Peng X., Liu G.X., Ren X.Q., Ziv O., Choi B.R. (2008). Mechanisms of cardiac arrhythmias and sudden death in transgenic rabbits with long QT syndrome. J. Clin. Invest..

[20] Liu G.X., Choi B.R., Ziv O., Li W., de Lange E., Qu Z. (2012). Differential conditions for early after-depolarizations and triggered activity in cardiomyocytes derived from transgenic LQT1 and LQT2 rabbits. J. Physiol..

[21] Terentyev D., Rees C.M., Li W., Cooper L.L., Jindal H.K., Peng X. (2014). Hyperphosphorylation of RyRs underlies triggered activity in transgenic rabbit model of LQT2 syndrome. Circ. Res..

[22] Dixon J.E., McKinnon D. (1994). Quantitative analysis of potassium channel mRNA expression in atrial and ventricular muscle of rats. Circ. Res..

[23] Wang T., Cheng Y., Dou Y., Goonesekara C., David J.P., Steele D.F. (2012). Trafficking of an endogenous potassium channel in adult ventricular myocytes. Am. J. Physiol. Cell Physiol..

[24] Decker M.L., Simpson D.G., Behnke M., Cook M.G., Decker R.S. (1990). Morphological analysis of contracting and quiescent adult rabbit cardiac myocytes in long-term culture. Anat. Rec..

[25] Decker M.L., Behnke-Barclay M., Cook M.G., Lesch M., Decker R.S. (1991). Morphometric evaluation of the contractile apparatus in primary cultures of rabbit cardiac myocytes. Circ. Res..

[26] Zhang Y., Li T.S., Lee S.T., Wawrowsky K.A., Cheng K., Galang G. (2010). Dedifferentiation and proliferation of mammalian cardiomyocytes. PLoS one.

[27] Tian Q., Pahlavan S., Oleinikow K., Jung J., Ruppenthal S., Scholz A. (2012). Functional and morphological preservation of adult ventricular myocytes in culture by sub-micromolar cytochalasin D supplement. J. Mol. Cell. Cardiol..

[28] Kabakov A.Y., Sengun E., Lu Y., Roder K., Bronk P., Baggett B. (2021). Three-week-old rabbit ventricular cardiomyocytes as a novel system to study cardiac excitation and EC Coupling. Front. Physiol..

[29] Wang Z., Feng J., Shi H., Pond A., Nerbonne J.M., Nattel S. (1999). Potential molecular basis of different physiological properties of the transient outward K+ current in rabbit and human atrial myocytes. Circ. Res..

[30] Patel S.P., Campbell D.L. (2005). Transient outward potassium current, 'Ito', phenotypes in the mammalian left ventricle: underlying molecular, cellular and biophysical mechanisms. J. Physiol..

[31] Brahmajothi M.V., Campbell D.L., Rasmusson R.L., Morales M.J., Trimmer J.S., Nerbonne J.M. (1999). Distinct transient outward potassium current (Ito) phenotypes and distribution of fast-inactivating potassium channel alpha subunits in ferret left ventricular myocytes. J. Gen. Physiol..

[32] Choi B.R., Li W., Terentyev D., Kabakov A.Y., Zhong M., Rees C.M. (2018). Transient outward K(+) current (Ito) underlies the right ventricular initiation of polymorphic ventricular tachycardia in a transgenic rabbit model of long-QT syndrome type 1. Circ. Arrhythm. Electrophysiol..

[33] Yau R., Rape M. (2016). The increasing complexity of the ubiquitin code. Nat. Cell Biol..

[34] Gao X., Gao S., Guan Y., Huang L., Huang J., Lin L. (2019). Toll-like receptor 3 controls QT interval on the electrocardiogram by targeting the degradation of Kv4.2/4.3 channels in the endoplasmic reticulum. FASEB J..

[35] Oudit G.Y., Kassiri Z., Sah R., Ramirez R.J., Zobel C., Backx P.H. (2001). The molecular physiology of the cardiac transient outward potassium current (I(to)) in normal and diseased myocardium. J. Mol. Cell. Cardiol..

[36] Roder K., Werdich A.A., Li W., Liu M., Kim T.Y., Organ-Darling L.E. (2014). RING finger protein RNF207, a novel regulator of cardiac excitation. J. Biol. Chem..

[37] Yuan L., Bu S., Du M., Wang Y., Ju C., Huang D. (2022). RNF207 exacerbates pathological cardiac hypertrophy via post-translational modification of TAB1. Cardiovasc. Res..

[38] An W.F., Bowlby M.R., Betty M., Cao J., Ling H.P., Mendoza G. (2000). Modulation of A-type potassium channels by a family of calcium sensors. Nature.

[39] Cercos P., Peraza D.A., Benito-Bueno A., Socuellamos P.G., Aziz-Nignan A., Arrechaga-Estevez D. (2021). Pharmacological approaches for the modulation of the potassium channelchannel KV4.x and KChIPs. Int. J. Mol. Sci..

[40] Deschenes I., DiSilvestre D., Juang G.J., Wu R.C., An W.F., Tomaselli G.F. (2002). Regulation of Kv4.3 current by KChIP2 splice variants: a component of native cardiac I(to)?. Circulation.

[41] Shibata R., Misonou H., Campomanes C.R., Anderson A.E., Schrader L.A., Doliveira L.C. (2003). A fundamental role for KChIPs in determining the molecular properties and trafficking of Kv4.2 potassium channels. J. Biol. Chem..

[42] Walsh I., Di Domenico T., Tosatto S.C. (2014). RUBI: rapid proteomic-scale prediction of lysine ubiquitination and factors influencing predictor performance. Amino Acids.

[43] Li Y., Xie P., Lu L., Wang J., Diao L., Liu Z. (2017). An integrated bioinformatics platform for investigating the human E3 ubiquitin ligase-substrate interaction network. Nat. Commun..

[44] Payapilly A., High S. (2014). BAG6 regulates the quality control of a polytopic ERAD substrate. J. Cell Sci..

[45] Buchberger A., Bukau B., Sommer T. (2010). Protein quality control in the cytosol and the endoplasmic reticulum: brothers in arms. Mol. Cell.

[46] Sturner E., Behl C. (2017). The role of the multifunctional BAG3 protein in cellular protein quality control and in disease. Front. Mol. Neurosci..

[47] Bhuin T., Roy J.K. (2015). Rab11 in disease progression. Int. J. Mol. Cell. Med..

[48] Moore D.J., West A.B., Dikeman D.A., Dawson V.L., Dawson T.M. (2008). Parkin mediates the degradation-independent ubiquitination of Hsp70. J. Neurochem..

[49] Kunjilwar K., Qian Y., Pfaffinger P.J. (2013). Functional stoichiometry underlying KChIP regulation of Kv4.2 functional expression. J. Neurochem..

[50] Wang H., Yan Y., Liu Q., Huang Y., Shen Y., Chen L. (2007). Structural basis for modulation of Kv4 K+ channels by auxiliary KChIP subunits. Nat. Neurosci..

[51] Bahring R., Dannenberg J., Peters H.C., Leicher T., Pongs O., Isbrandt D. (2001). Conserved Kv4 N-terminal domain critical for effects of Kv channel-interacting protein 2.2 on channel expression and gating. J. Biol. Chem..

[52] Connell P., Ballinger C.A., Jiang J., Wu Y., Thompson L.J., Hohfeld J. (2001). The co-chaperone CHIP regulates protein triage decisions mediated by heat-shock proteins. Nat. Cell Biol..

[53] Okiyoneda T., Barriere H., Bagdany M., Rabeh W.M., Du K., Hohfeld J. (2010). Peripheral protein quality control removes unfolded CFTR from the plasma membrane. Science.

[54] Imai Y., Soda M., Hatakeyama S., Akagi T., Hashikawa T., Nakayama K.I. (2002). CHIP is associated with Parkin, a gene responsible for familial Parkinson's disease, and enhances its ubiquitin ligase activity. Mol. Cell.

[55] Tsuji Y. (2020). Transmembrane protein western blotting: impact of sample preparation on detection of SLC11A2 (DMT1) and SLC40A1 (ferroportin). PLoS one.

[56] Manganas L.N., Trimmer J.S. (2000). Subunit composition determines Kv1 potassium channel surface expression. J. Biol. Chem..

