# Supplementary material for: E3 ubiquitin ligase rififylin has yin and yang effects on rabbit cardiac transient outward potassium currents (Ito) and corresponding channel proteins
Source: J Biol Chem. 2024 Feb 15;300(3):105759. doi: 10.1016/j.jbc.2024.105759 (PMC10945274; doi:10.1016/j.jbc.2024.105759)
Supplement: Supporting Figures S1–S13 [file mmc1.pdf]

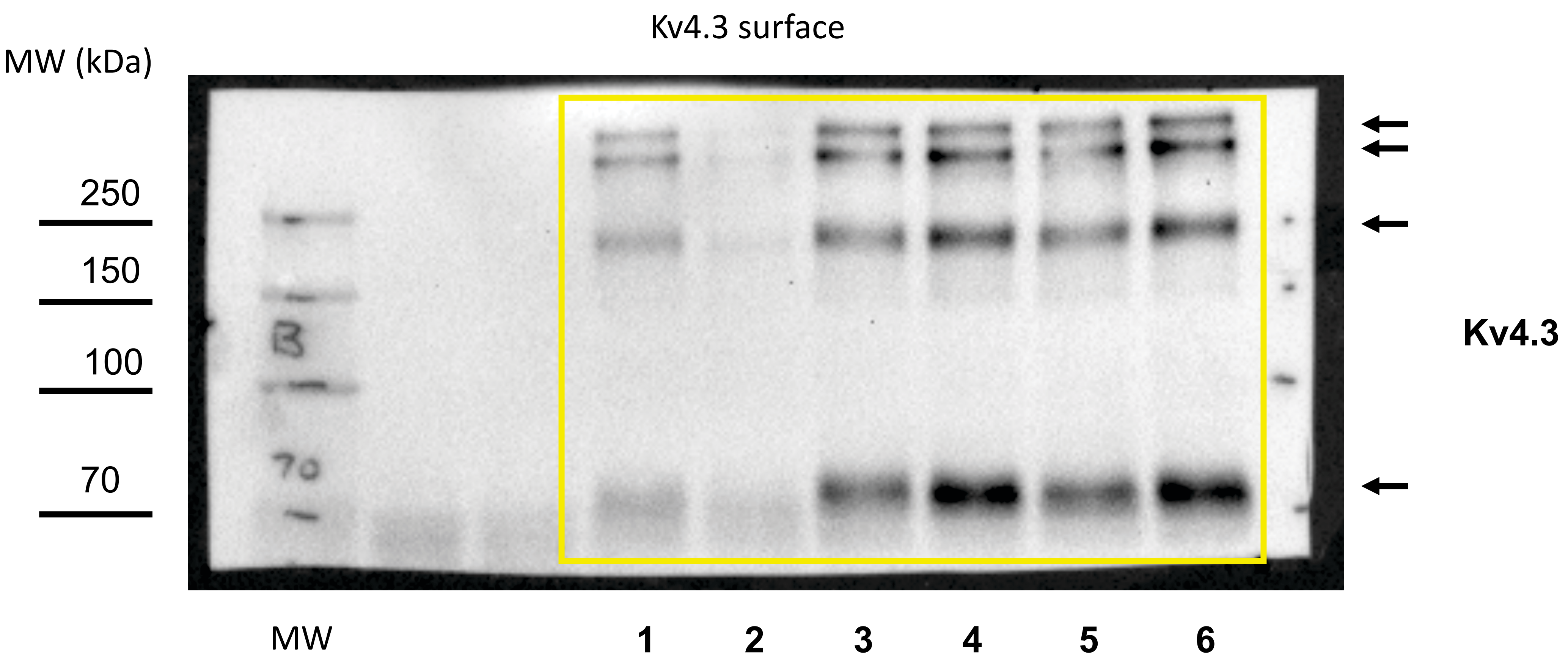

Original uncropped immunoblot image for Kv4.3 surface expression. Bands 1-6 boxed in yellow are used in Figure 3A.

Supplemental Figure 1

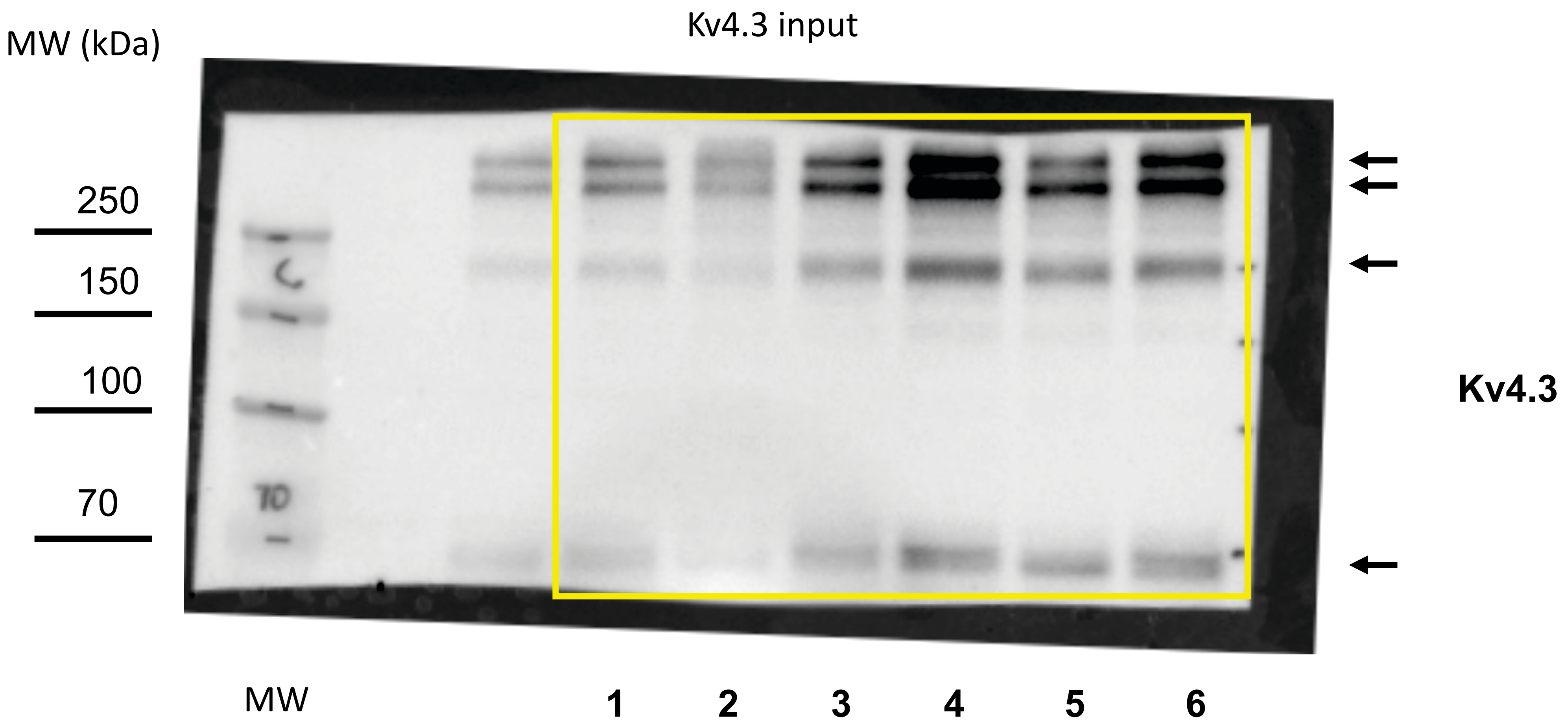

Original uncropped immunoblot image for Kv4.3 total expression. Bands 1-6 boxed in yellow are used in Figure 3A.

Supplemental Figure 2

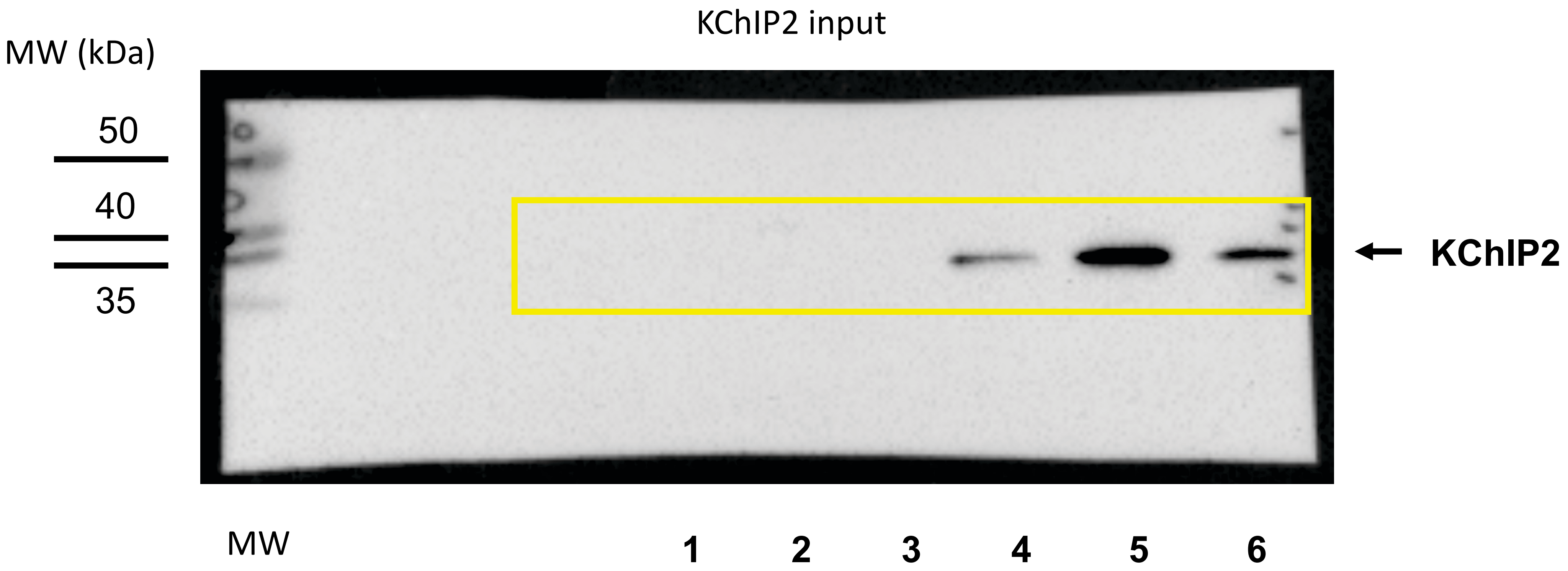

Original uncropped immunoblot image for KChIP2 total expression. Bands 1-6 boxed in yellow are used in Figure 3A.

Supplemental Figure 3

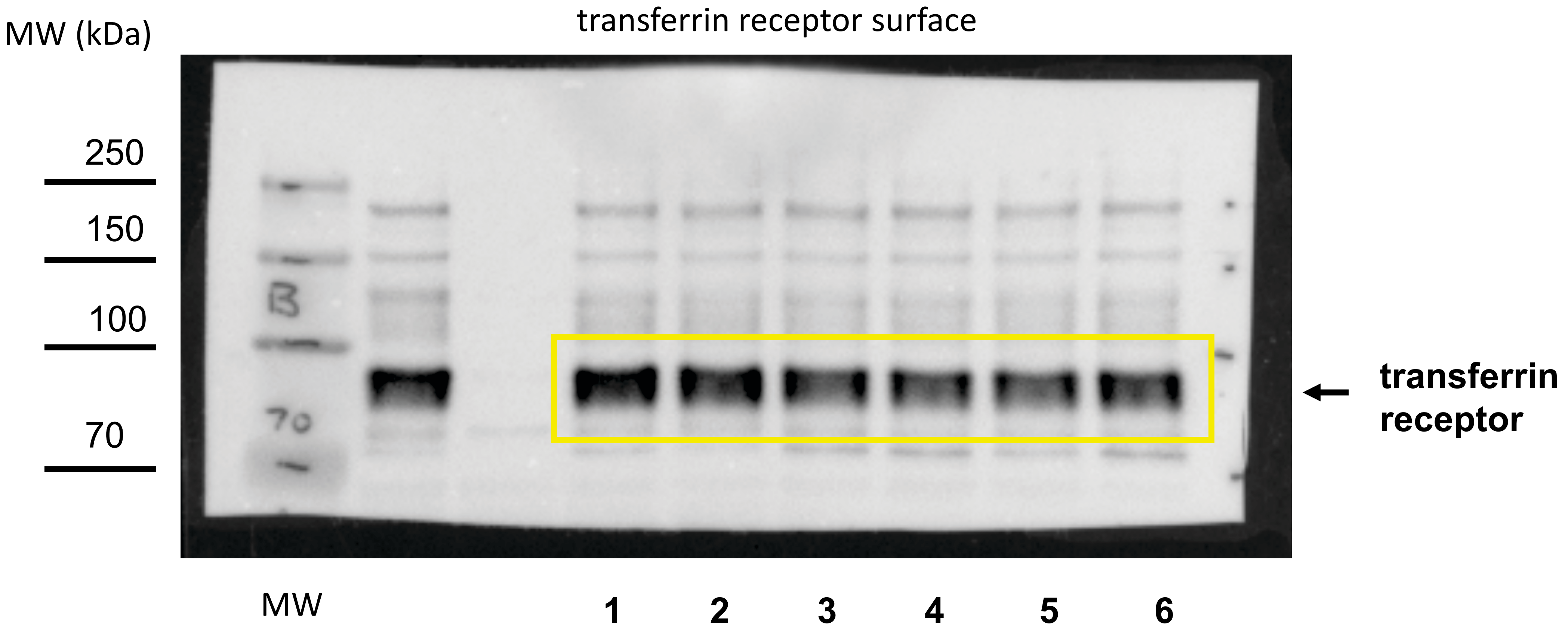

Original uncropped immunoblot image for transferrin receptor surface expression. Bands 1-6 boxed in yellow are used in Figure 3A.

Supplemental Figure 4

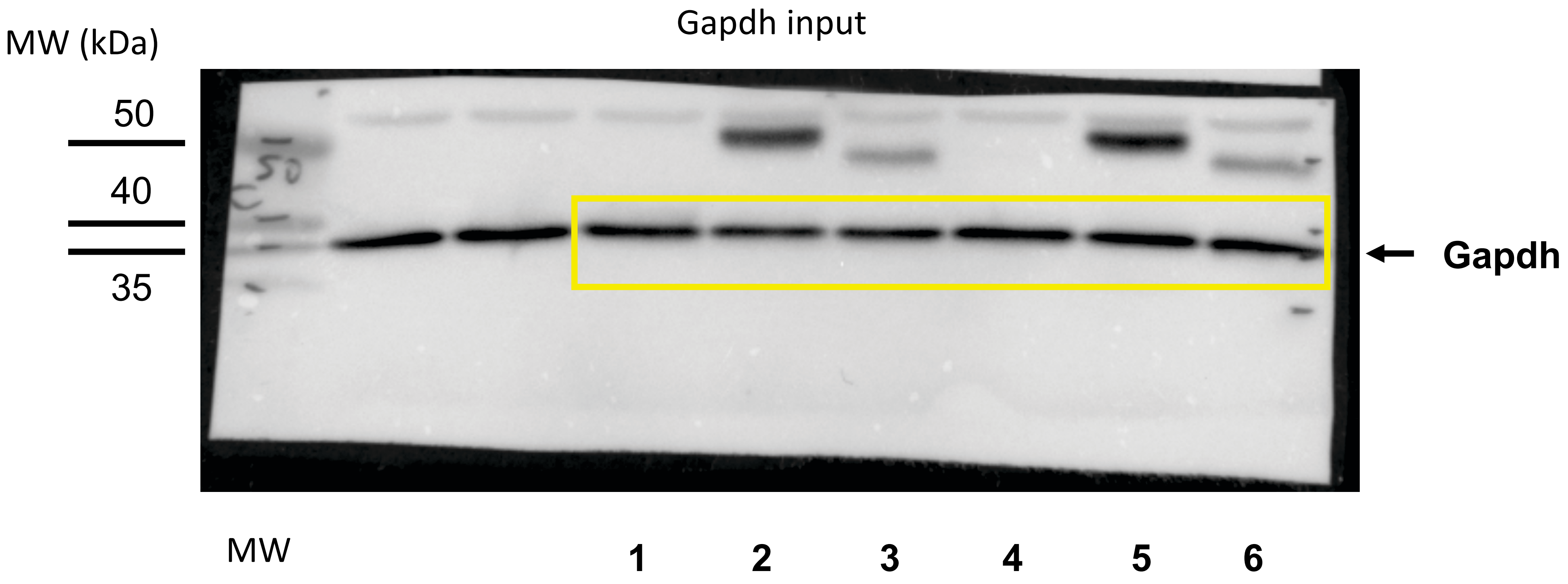

Original uncropped immunoblot image for Gapdh total expression. Bands 1-6 boxed in yellow are used in Figure 3A.

Supplemental Figure 5

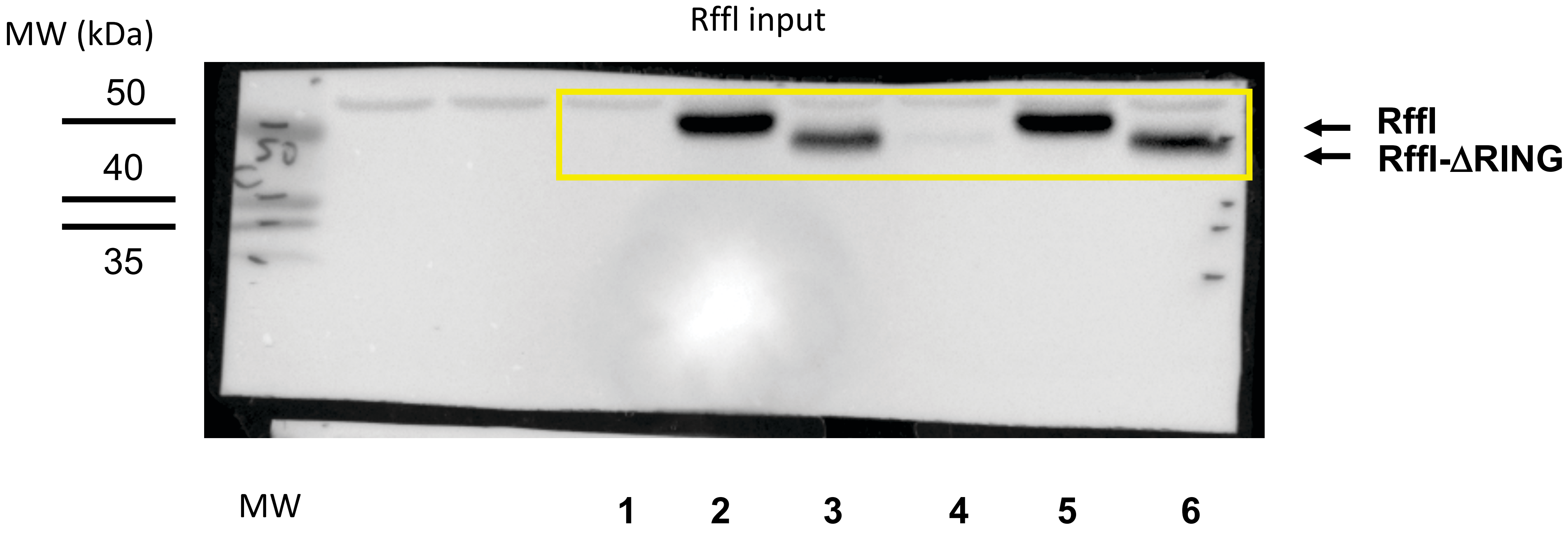

Original uncropped immunoblot image for Flag-tagged Rffl/Rffl-ΔRING total expression. Bands 1-6 boxed in yellow are used in Figure 3A.

Kv1.4 surface input

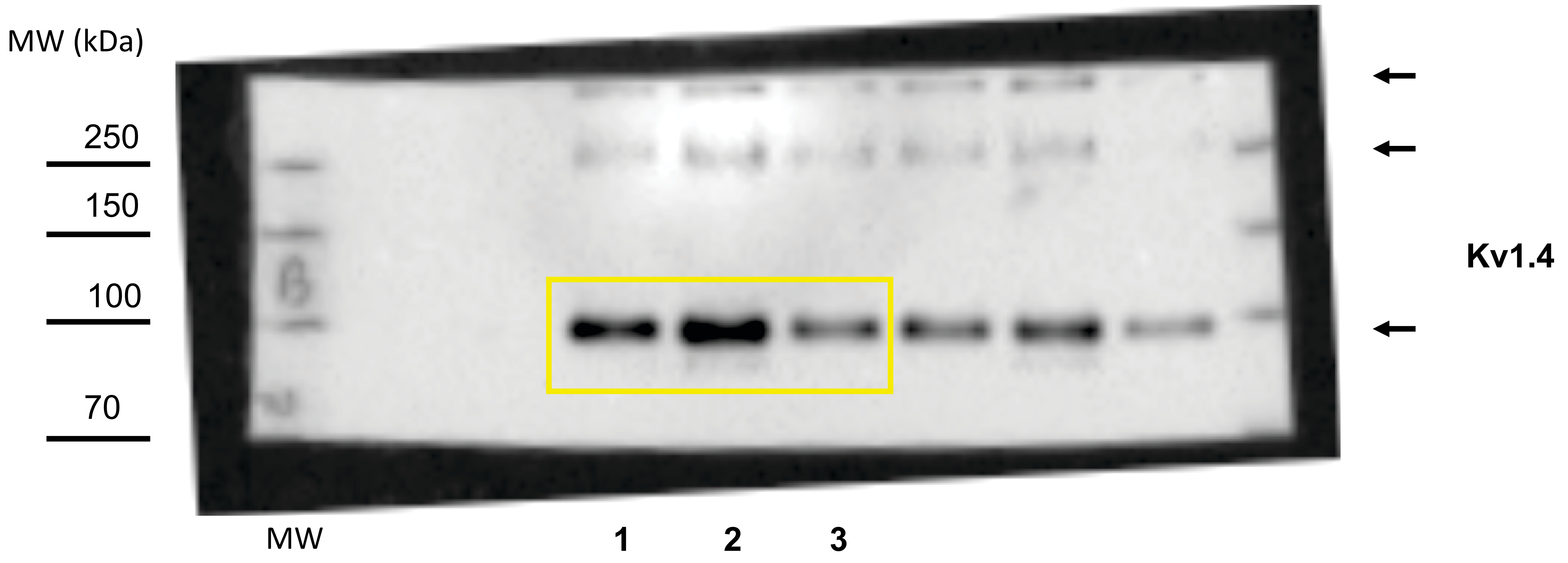

Original uncropped immunoblot image for Kv1.4 surface expression. Bands 1-3 boxed in yellow are used in Figure 5A.

Supplemental Figure 7

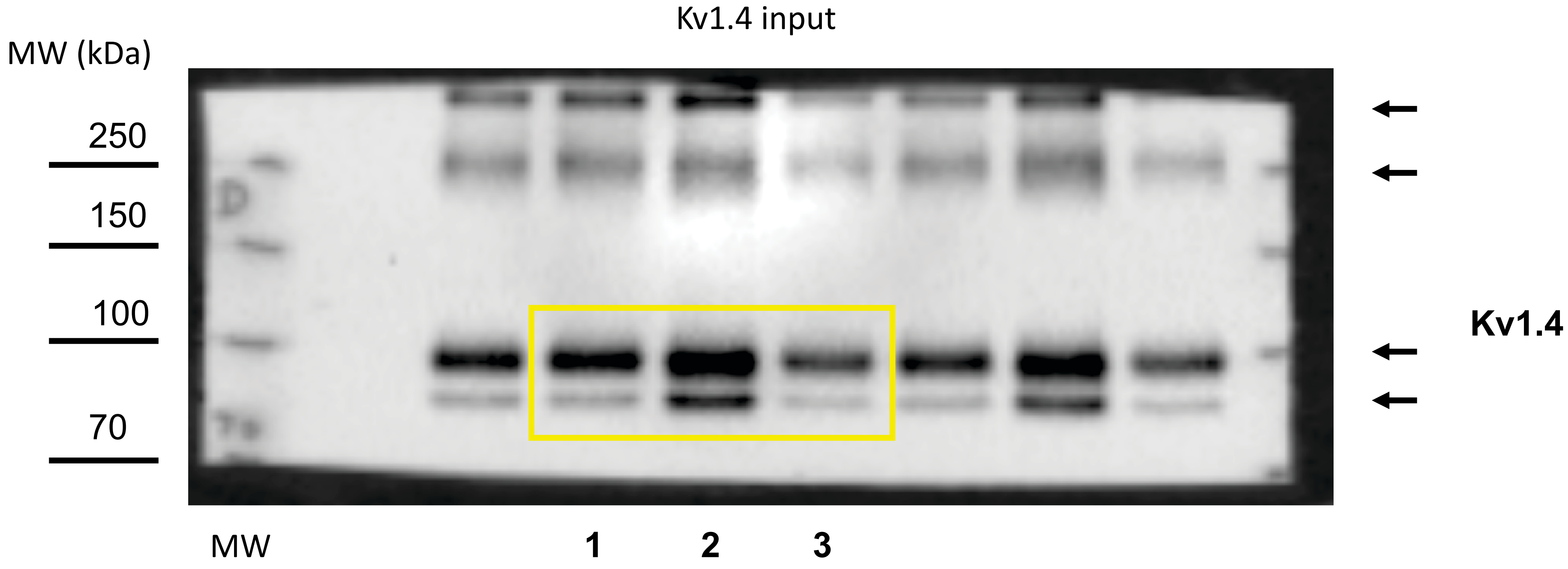

Original uncropped immunoblot image for Kv1.4 total expression. Bands 1-3 boxed in yellow are used in Figure 5A.

Supplemental Figure 8

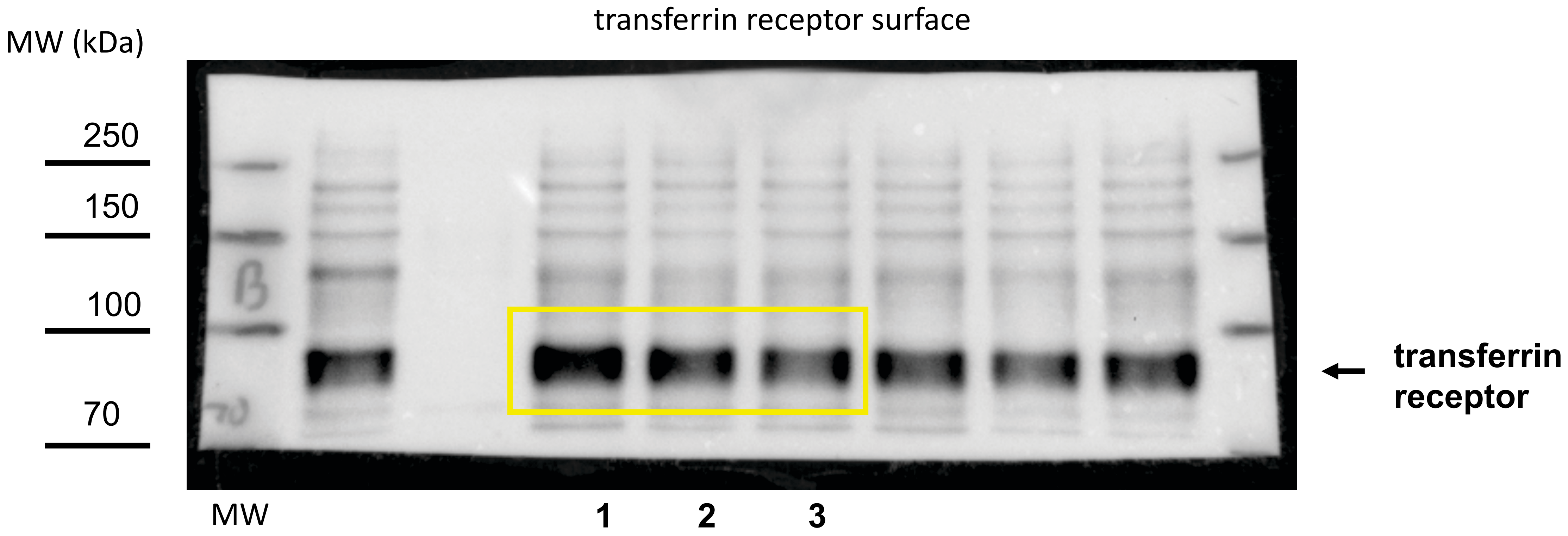

Original uncropped immunoblot image for transferrin receptor surface expression. Bands 1-3 boxed in yellow are used in Figure 5A.

Supplemental Figure 9

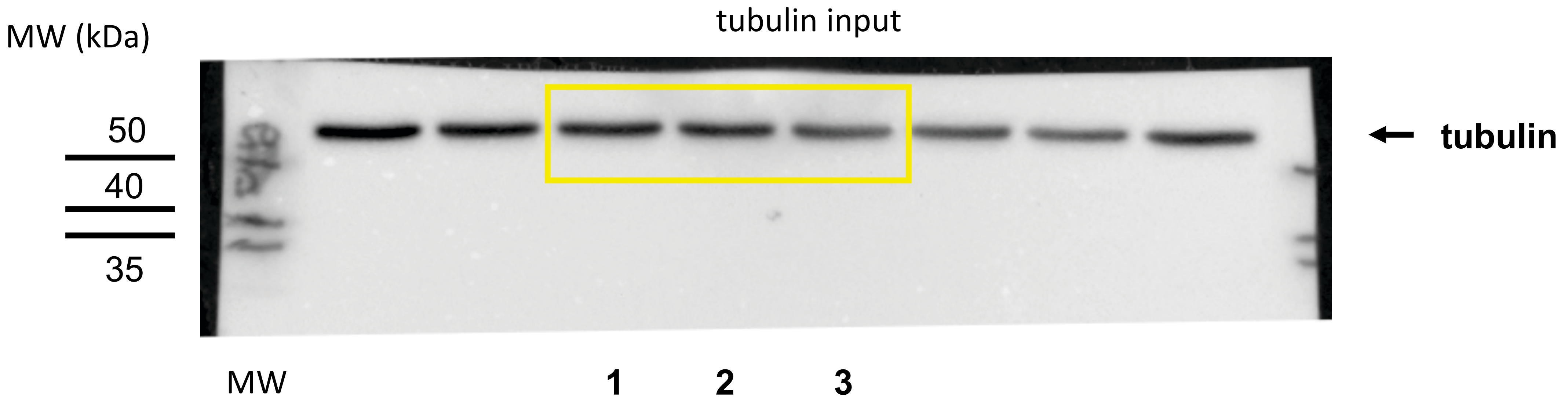

Original uncropped immunoblot image for alpha tubulin total expression. Bands 1-3 boxed in yellow are used in Figure 5A.

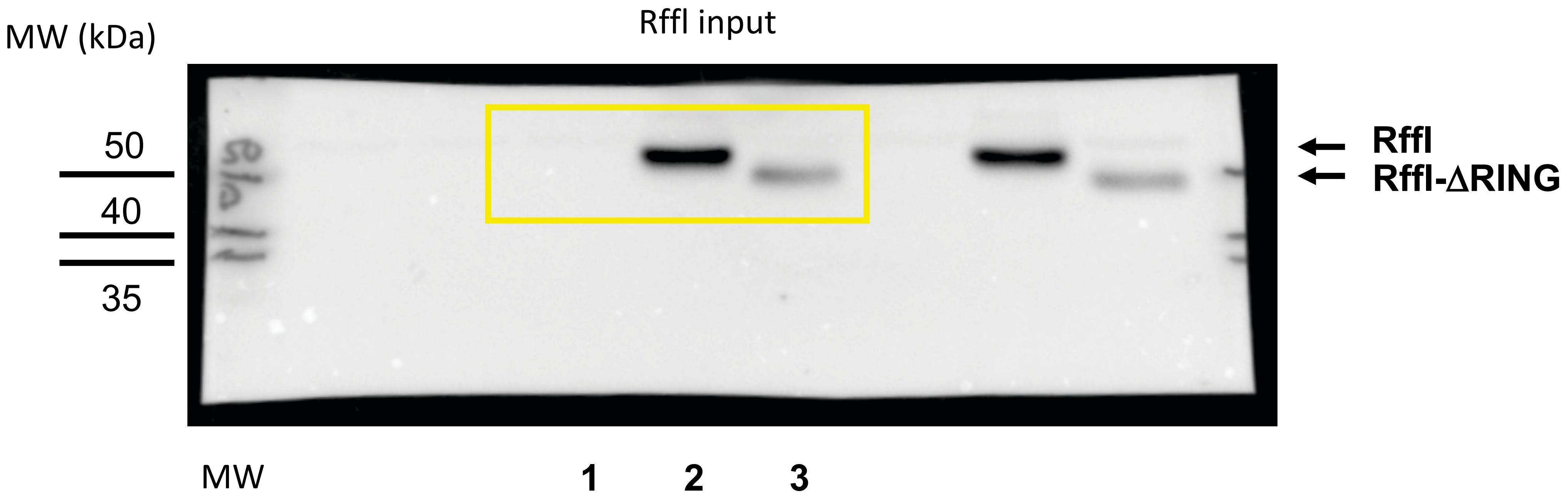

Original uncropped immunoblot image for Flag-tagged Rffl/Rffl-ΔRING total expression. Bands 1-3 boxed in yellow are used in Figure 5A.

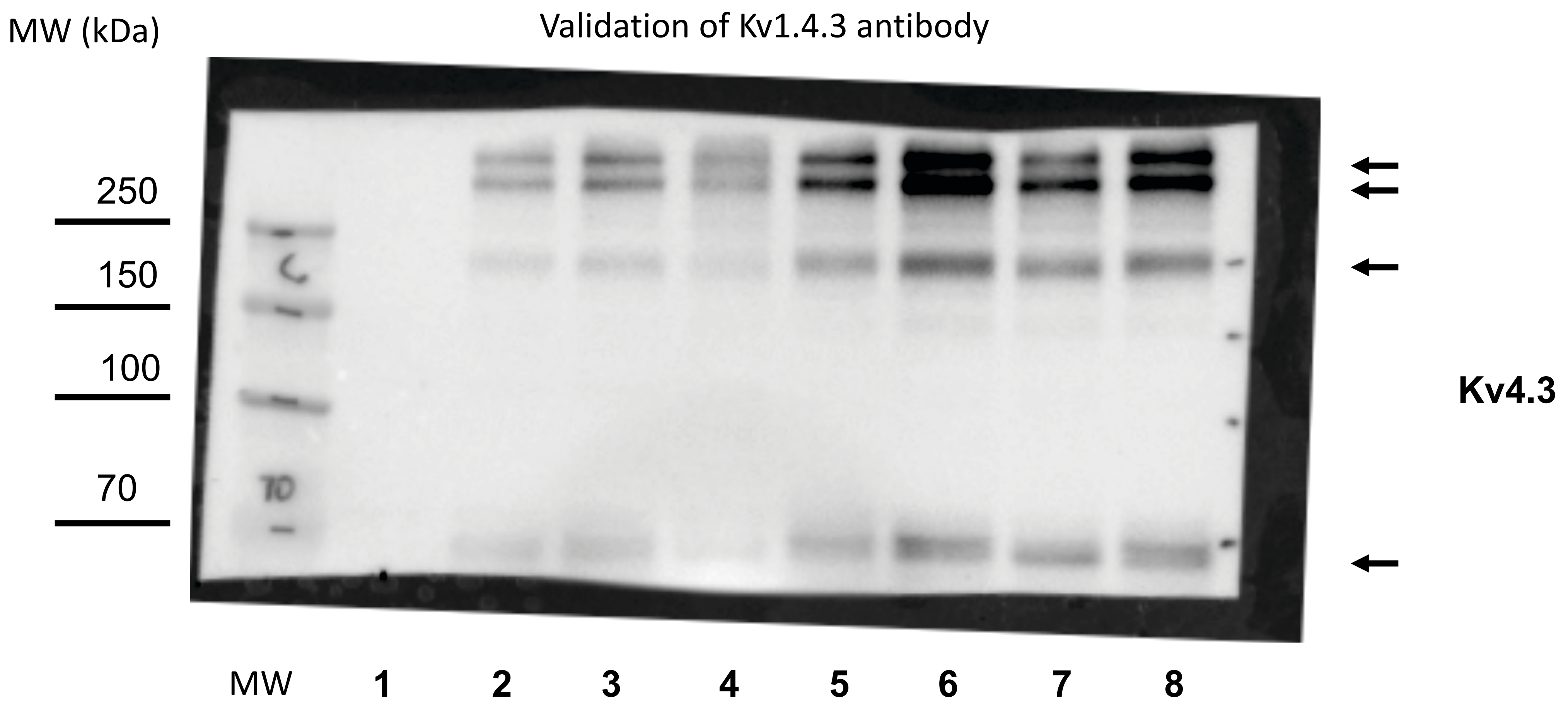

Original uncropped immunoblot image for Kv4.3 total expression used for surface biotinylation experiments (see supplemental figure 2) to validate Kv4.3 antibody (rabbit anti-Kv4.3 [Chemicon; AB5194; 1:1,000]). Lane 1: non-transfected; lanes 2, 3, and 6: transfection with Kv4.3 plasmid; lanes 4 and 7: transfection with Kv4.3 and Rffl plasmids; and lanes 5 and 8: transfection with Kv4.3 and Rffl-ΔRING plasmids. Arrows indicate Kv4.3 signals.

Supplemental Figure 12

Validation of Kv1.4 antibody

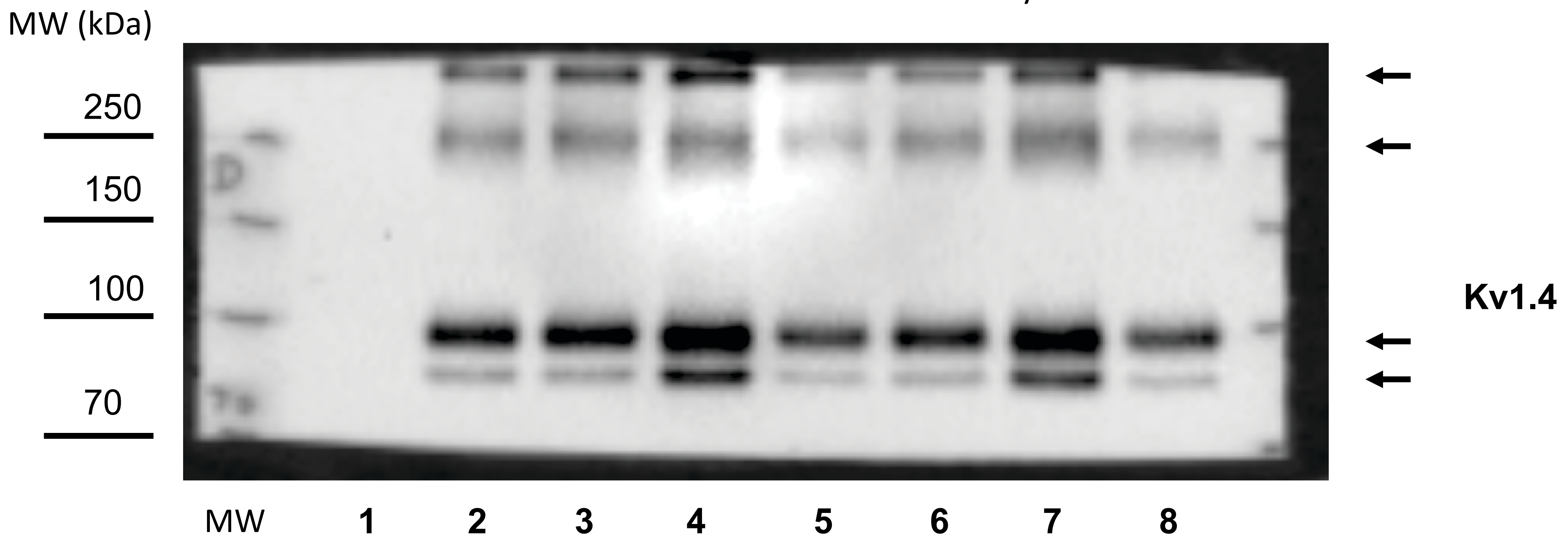

Original uncropped immunoblot image for Kv1.4 total expression used for surface biotinylation experiments (see supplemental figure 8) to validate Kv1.4 antibody (mouse anti-Kv1.4 [UC Davis/NIH NeuroMab Facility; AB_2877317; 1:1,000]). Lane 1: non-transfected; lanes 2, 3, and 6: transfection with Kv1.4 plasmid; lanes 4 and 7: transfection with Kv1.4 and Rffl plasmids; and lanes 5 and 8: transfection with Kv1.4 and Rffl-ΔRING plasmids. Arrows indicate Kv1.4 signals.

Supplemental Figure 13
