# Supplementary material for: E3 ubiquitin ligase rififylin has yin and yang effects on rabbit cardiac transient outward potassium currents (Ito) and corresponding channel proteins
Source: J Biol Chem. 2024 Feb 15;300(3):105759. doi: 10.1016/j.jbc.2024.105759 (PMC10945274; doi:10.1016/j.jbc.2024.105759)
Supplement: Table S1 [file mmc2.docx]

**Table S1.** RFFL has no significant effects on *I*_to_ kinetics in rabbit cardiomyocytes (RbCMs). However, RFFL has an effect on the relative contributions of *I*_to,f_ and *I*_to,s_ amplitudes to the total *I*_to_.

| ***I*_to_ parameters in RbCMs** | **GFP** | **RFFL** |
| --- | --- | --- |
| *I*_to_ (A/F) | 8.7±1.0 | 8.3±1.6 |
| Fast inactivation (ms) | 22±1.5 | 19±0.8 |
| Slow inactivation (ms) | 180±16 | 218±58 |
| Fast recovery (%) | 35±3 | **21±2*** |
| Slow recovery (%) | 65±3 | **79±2*** |
| ***** - significant effect of RFFL (p < 0.005) | | |
