# Supplementary material for: E3 ubiquitin ligase rififylin has yin and yang effects on rabbit cardiac transient outward potassium currents (Ito) and corresponding channel proteins
Source: J Biol Chem. 2024 Feb 15;300(3):105759. doi: 10.1016/j.jbc.2024.105759 (PMC10945274; doi:10.1016/j.jbc.2024.105759)
Supplement: Table S2 [file mmc3.docx]

**Table S2.** There is no significant RFFL effect on *I*_to_ kinetics in HEK cells expressing either Kv4.3 and KChIP2 together, or Kv1.4 alone.

| ***I*_to_ kinetics in HEK cells** | **GFP** | **RFFL** |
| --- | --- | --- |
| Kv4.3 fast inactivation (ms) | 16.9±0.7 | 15.0±1.3 |
| Kv4.3 slow inactivation (ms) | 77±15 | 87±23 |
| Kv1.4 fast inactivation (ms) | 19.2±1.2 | 18.5±0.8 |
| Kv1.4 slow inactivation (ms) | 226±10 | 211±11 |
