# Supplementary material for: E3 ubiquitin ligase rififylin has yin and yang effects on rabbit cardiac transient outward potassium currents (Ito) and corresponding channel proteins
Source: J Biol Chem. 2024 Feb 15;300(3):105759. doi: 10.1016/j.jbc.2024.105759 (PMC10945274; doi:10.1016/j.jbc.2024.105759)
Supplement: Table S3 [file mmc4.docx]

**Table S3.** Computer simulation of rabbit ventricular myocytes with ADP90 values at various stimulation cycle lengths (CL) and application of isoproterenol (ISO) in control cells (GFP) and at various changes in *I*_Kr_, *I*_Ks_, *I*_to,f_ and *I*_to,s_ currents. The very right column corresponds to the RFFL effect on top of LQT2 phenotype.

| Cycle length | **GFP** | ***I*_Kr_ = 0** | **LQT2: *I*_Kr_ = 0; 70% *I*_Ks_** | **LQT2: *I*_Kr_ = 0; 70% *I*_Ks_; & RFFL *I*_to_: g_to,f_ = 0.032, g_to,s_ = 0.122 (mS/μF)** |
| --- | --- | --- | --- | --- |
| CL = 0.4 s | 217 | 237 | 266 | 258 |
| CL = 4 s | 284 | 306 | 349 | 349 |
| CL = 4 s, ISO | 322 | 347 | 416 | 403 |
